# Effects of blood flow restriction training on physical fitness among athletes: a systematic review and meta-analysis

**DOI:** 10.1038/s41598-024-67181-9

**Published:** 2024-07-18

**Authors:** Kun Yang, Chen Soon Chee, Johan Abdul Kahar, Tengku Fadilah Tengku Kamalden, Rui Li, Shaowen Qian

**Affiliations:** 1 Department of Sports Studies, Faculty of Educational Studies, Universiti Putra Malaysia, Selangor, Malaysia; 2 Department of Orthopedics, Faculty of Medicine and Health Sciences, Universiti Putra Malaysia, Selangor, Malaysia; 3 National Sports Institute, National Sports Complex, Kuala Lumpur, Malaysia; 4 Department of Physical Education, Wuhan Sports University, Wuhan, China

**Keywords:** Health care, Public health

## Abstract

Blood flow restriction training (BFRT) is an effective, scientific and safe training method, but its effect on the overall quality of athletes remains unclear. The aim of this systematic review with meta-analysis was to clarify the effects of BFRT on the physical fitness among athletes. Based on the PRISMA guidelines, searches were performed in PubMed, Web of Science, SPORTDiscus, and SCOUPS, the Cochrane bias risk assessment tool was used to assess methodological quality, and RevMan 5.4 and STATA 15.0 software were used to analyze the data. A meta-analysis of 28 studies with a total sample size of 542 athletes aged 14–26 years and assessed as low risk for quality was performed. Our results revealed that the BFRT intervention had small to large improvements in the athletes' strength (ES = 0.74–1.03), power (ES = 0.46), speed (ES = 0.54), endurance (ES = 1.39–1.40), body composition (ES = 0.28–1.23), while there was no significant effect on body mass (p > 0.05). Subgroup analyses revealed that moderator variables (training duration, frequency, load, cuff pressure, and pressurization time) also had varying degrees of effect on athletes' physical fitness parameters. In conclusion, BFRT had a positive effect on the physical fitness parameters of the athletes, with significantly improved strength, power, speed, endurance and body composition, but not body mass parameters. When the training frequency ≥ 3 times/week, cuff pressure ≥ 160 mmHg, and pressurization time ≥ 10 min, the BFRT group was more favorable for the improvement of physical fitness parameters.

## Introduction

Athlete level is determined by the integration of physical fitness, technique, tactics, psychology, and game performance^1^. Physical fitness refers to all aspects of an athlete's well-being and sports ability^2,3^. As is well known, strength, speed, endurance, agility, body composition and balance of physical fitness play an important role in intense competition^3,4^. Notably, athletes' physical fitness is intimately related to their sports performance, since any one of the parameter characteristics of physical fitness can correspond to at least one of the sports skill indicators, such as tackling and defensive skills correspond to power, speed and agility, followed by high level of sports skills are more beneficial to have better sports performance in the game, so improving the physical fitness can promote the maximum transfer of sports skills to sports performance^4–6^. In addition, athletes are more susceptible to suffering some degree of sports injury during long-term training with excessive loads (e.g., the mechanical load and/or the volume), whereas professionally supervised resistance training can prevent and/or rehabilitate injuries^7,8^. More specifically, when athletes complete their daily training volume at traditionally high loads, additional supplemental low-load BFRT (LL-BFRT) can induce similar morphological adaptations and strength gains while reducing total loads compared to high-load resistance training (HL-RT), resulting in effective injury prevention^9^. Similarly, when the rehabilitating population performs strength exercises, LL-BFRT can reduce the stresses and loads on injured joints and soft tissues compared to traditional HL-RT, thereby reducing the risk of injury during the exercise^10^.

In the past, studies have confirmed that resistance training in a conditioning program alters physical fitness components^11^. The American College of Sports Medicine states that traditional resistance training increases fitness levels^12^. Nevertheless, traditional resistance training modalities primarily use large weights and low repetitions (70–90% of 1-repetition maximum (1RM), 8–12 reps per set), which can easily lead to training injuries and are not applicable to the rehabilitation of the joint-injured population^3,8^. BFRT primarily uses small weights and high repetitions (20–40% 1RM, 15–30 reps per set) that can be effective for improving muscle strength and mass for rehabilitation groups or athletes^13^. Therefore, BFRT has gained importance as an efficient, scientific and safe training method in the fields of rehabilitation training^8^, fitness^14^, and sports training^15^.

BFRT is a method of applying a specific pressurized cuff to the proximal limbs to modulate blood flow, which can be used alone or in combination with different resistance modalities^16^. Due to the restriction of blood flow, the muscles create a hypoxic and ischemic internal environment during BFRT possibly increasing neuromuscular adaptations such as improvements in morphology (e.g., muscle mass and tendon stiffness) and performance (e.g., muscular strength and endurance)^17–19^. Moreover, LL-BFRT can achieve on equal neuromuscular adaptations (especially morphologic and neuronal adaptations) as traditional HL-RT, but induces lower muscle swelling and soreness after BFRT, thereby favoring faster recovery and thus allowing for long-term scheduling during the physical training cycle^20–23^.

Currently, the effectiveness of BFRT has been confirmed by a large number of experts and scholars, including in the area of improving physical fitness^24,25^. More specifically, BFRT can be more effective for improving an athlete's power (e.g., vertical jump performance), speed (e.g., 10 m, 20 m sprints), and endurance (e.g., maximal oxygen consumption), as compared to traditional resistance training^24,26^. Furthermore, studies have reported benefits of BFRT for both physical confrontational (e.g., soccer) and non-confrontational athletes (e.g., gymnastics)^27,28^. The current systematic review only addresses the study of a single indicator or multiple indicators without synthesizing all of them. Therefore, the aim of this systematic review with meta-analysis was to assess the effect of BFRT on physical fitness parameters of athletes, as well as analyzed the impact of potential moderators on outcomes.

## Methods

### Protocol and registration

This meta-analysis was performed following the PRISMA guidelines^29^, and this meta-analysis was registered on inplasy.com (INPLASY202320040).

### Search strategy

The literature search was undertaken in the PubMed, Web of Science, SPORTDiscus, and SCOUPS, whereas the search timeline for the included studies ranged from the earliest record to May 2024. Boolean algorithms are utilized in this study. Each database was searched by title using a predefined combination of keywords: (“blood flow restriction” OR “occlusive training” OR “vascular occlusion” OR “kaatsu” OR “ischemia”) AND (“physical fitness” OR “strength” OR “power” OR “speed” OR “endurance” OR “agility” OR “flexibility” OR “balance” OR “body composition” OR “coordination” OR “anaerobic” OR “aerobic” OR “cardiorespiratory capacity” OR “skill-related fitness” OR “physical conditioning”) AND (“athlete” OR “player” OR “sportsperson”). Furthermore, supplementary searches were conducted using Google Scholar as well as other relevant papers in the research reference list.

### Eligibility criteria

As shown in Table 1, the PICOS criteria have been used as inclusion and exclusion criteria for this research. Only BFRT records in terms of physical fitness in healthy athletes were included in the meta-analysis. The eligibility criteria were as follows: (1) Peer-reviewed publications in full English. In addition, the subjects are healthy athletes regardless of gender, age or sport restrictions; (2) Studies had full training protocols for blood flow restriction (BFR) interventions, in which BFRT or BFR combined with other load training interventions were used; (3) The study design was a RCT as well as a two-group or multi-group trial using pre-test and post-test; (4) At least one physical fitness-related measure (e.g., strength) is reported in the article.

**Table 1:** Eligibility criteria for inclusion in the study. BFR, blood flow restriction; RCT, randomized controlled trials.

Category	Inclusion criteria	Exclusion criteria
Population	Athletes, male or female, any sports activity, no age restriction	Athletes with health problems (injury or nearby surgery) and Interference factors
Intervention	BFRT (BFR combined with other forms of training)	Without BFR
Comparison	Two-group or multi-group trials	Single-group trials
Outcome	At least one measure related to physical fitness (e.g., strength)	No physical fitness data
Study design	RCT	Non-RCT

### Study selection and data extraction

**Figure 1:**
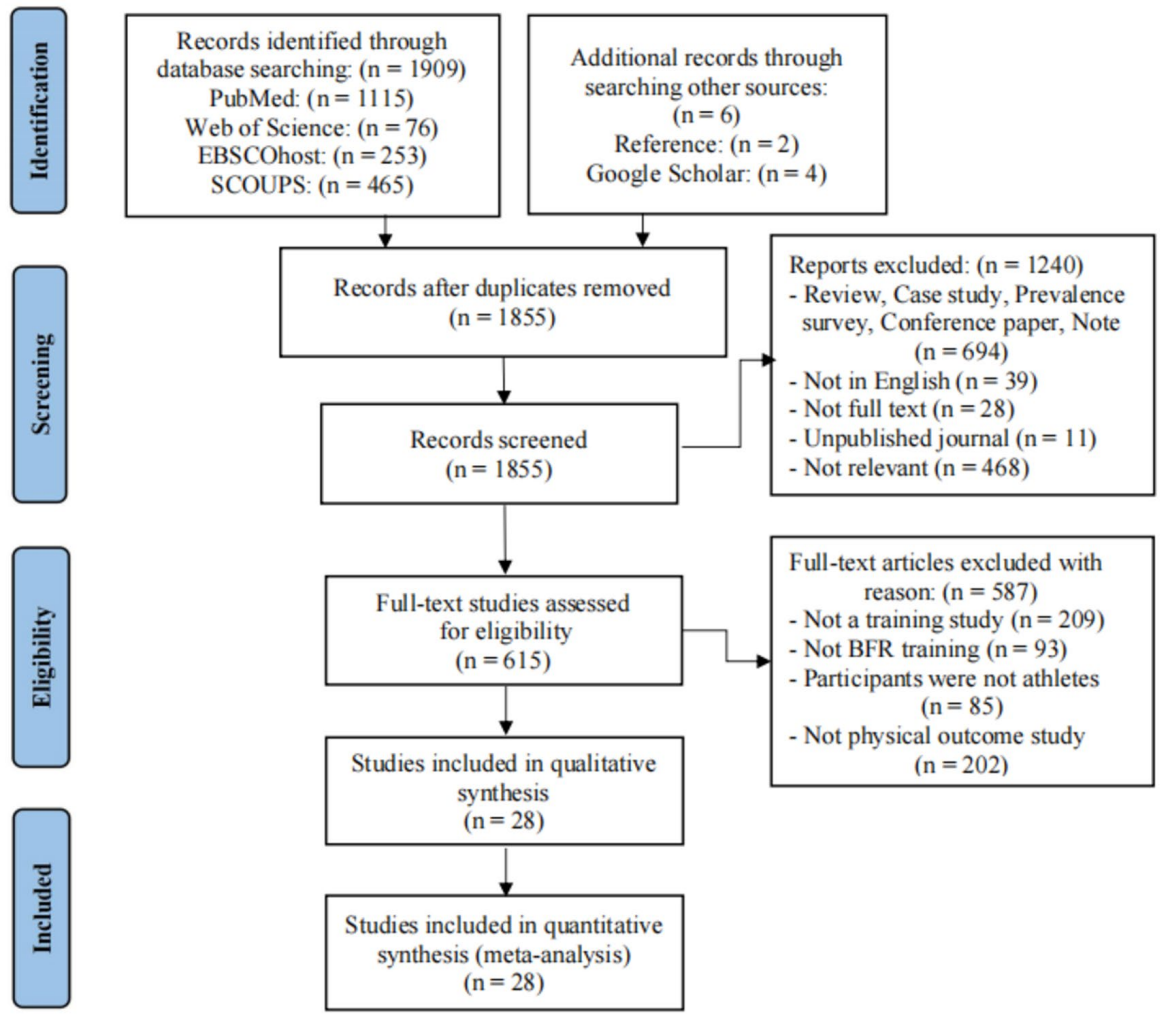
Flow diagram of the search process.

Two authors (KY, RL) searched through the electronic database and uploaded to the EndNodeX9 reference management software, which allowed one-click screening of duplicate articles. The title and abstract were then used to further screen the articles. The next step was the reading of the entire text and the inclusion of the articles that were in accordance with the eligibility criteria after exclusion. In addition, when two authors disagreed on screening, the authors (CSC) were immediately consulted until a consensus view was in place. The whole screening and exclusion process is shown in Fig. 1.

After search and screen out by both authors, and after recording the following study data: (1) Authors, title, and publication date; (2) Number, gender, age, height, body mass, training experience, sports and fitness level of study subjects; (3) Intervention characteristics of BFRT including frequency, duration, load, protocol, cuff location and width, pressure, pressurization time and status; (4) Study design, comparison, and outcomes.

### Quality assessment

Two authors assessed the methodological quality of the screened articles using the Cochrane Bias Risk Assessment Tool (RevMan 5.4)^30^. The assessment tool contains six aspects, namely selection bias, performance bias, detection bias, attrition bias, reporting bias, and other biases, as well as three risk ratings. However, when two authors disagreed on the assessment scores, the authors (CSC) were immediately consulted until a consensus view was in place.

### Statistical analysis

The meta-analysis was performed using RevMan version 5.4 software. However, based on previous studies, meta-analyses were performed only on data with ≥ 3 studies of the same physical fitness parameters^31^. In addition, effect sizes (ES) were estimated based on the sample size, mean and standard deviation before and after the intervention. ES is expressed as standardized mean difference (SMD) with 95% confidence interval (small, < 0.6; moderate, 0.6–1.2; large, > 1.2)^32^. Furthermore, the I^2^ statistic was used to assess the inter-study heterogeneity (low, < 25%; medium, 25–75%; high, > 75%)^33^. Different heterogeneity matched with different effect models (low, fixed; high, random)^34^. Effect models were used to explain between-group differences that may affect BFR effects^35^. Immediately after, we used STATA 15.0 software for sensitivity analysis to assess the robustness of the results, as well as publication bias assessment using funnel plots. Statistical significance was determined at p < 0.05.

### Additional analyses

Subgroup analyses further explored potential moderators of BFRT that may affect outcomes. Moderators in the training intervention characteristics included the BFRT duration (≤ 6 vs. > 6 weeks), frequency (< 3 vs. ≥ 3 times/week), load (low, < 50% 1RM or maximal heart rate or heart rate reserve; moderate, 50–70%; high, > 70%)^12,13,36^, cuff pressure (< 160 vs. ≥ 160 mmHg), and pressurization time (< 10 vs. ≥ 10 min). Moreover, each moderator must satisfy at least 3 studies and calculated by median splitting technique^37^.

## Results

### Study selection

The records identified by the two authors through database searches were 1909 articles, including 1115 in PubMed, 76 in Web of Science, 253 in SPORTDiscus, and 465 in SCOUPS, as well as 2 in Refereed and 4 in Google Scholar. After eliminating the duplicate 60 articles using EndNodeX9 reference management software, 1855 unduplicated articles remained. Therefore, after screening out 1827 articles according to the eligibility criteria, the remaining 28 articles finally met the inclusion criteria for this study. These articles were published between 2000 and 2023, as shown in Fig. 1.

### Characteristics of included studies

The 28 articles included in this meta-analysis included 542 healthy athletes, 274 physically confrontational and 268 non-confrontational. The mean age of all athletes was 21 years, height ranged from 152.4 to 195.4 cm and weight ranged from 43.1 to 99.1 kg, 20 articles analyzed male athletes, 2 articles analyzed female athletes and 5 articles analyzed both male and female athletes, with at least 0.5 year of training experience, and trained or highly trained (see Table 2).

**Table 2:** Characteristics of included study participants.

References	Athletes	N	Age (years)	Gender	Height (cm)	Weight (kg)	TE ( years )	SFL
Takarada et al., 2002^38^	Rugby	12	25.3/26.3	M	179.3/181.0	88.9/92.4	≥ 5	Tier 2
Abe et al., 2005^39^	Track & Field	15	NR	M	173.9/176.8	66.1/67.6	NR	Tier 2
Sakuraba et al., 2009^40^	Track & Field	12	20.0/19.9	M	172.2/178.6	66.0/66.8	NR	Tier 2
Park et al., 2010^19^	Basketball	12	20.1/20.8	M	186.1/192.6	83.9/92.4	NR	Tier 3
Yamanaka et al., 2012^9^	Soccer	32	19.2 ± 1.8	M	181.8/181.1	91.3/89.7	≥ 5	Tier 3
Godawa et al., 2012^41^	Powerlifter	18	21.0/22.0	M/F	177.1/173.5	89.9/77.5	≥ 1	Tier 2
Manimmanakorn et al., 2013a^24^	Netball	20	20.2 ± 3.3	F	168.4 ± 5.8	65.2 ± 6.5	NR	Tier 2
Manimmanakorn et al., 2013b^42^	Netball	20	20.2 ± 3.3	F	168.4 ± 5.8	65.2 ± 6.5	NR	Tier 2
Cook et al., 2014^43^	Rugby	20	21.8/21.1	M	184.0/184.0	94.7/96.4	≥ 2	Tier 3
Luebbers et al., 2014^44^	Soccer	31	20.3 ± 1.1	M	NR	99.1 ± 19.7	≥ 5	Tier 2
Scott et al., 2017^26^	Soccer	18	19.8 ± 1.5	M	186.0 ± 8.0	80.8 ± 8.2	NR	Tier 3
Behringer et al., 2017^45^	Runner	24	25.6/21.7	M	181.4/181.2	79.1/76.1	NR	Tier 2
Amani et al., 2018^46^	Soccer	19	23.9 ± 2.3	M	176.1 ± 4.1	73.0 ± 3.9	≥ 7	Tier 2
Luebbers et al., 2019^47^	Powerlifter	17	15.8/16.6	M/F	179.5/177.9	74.8/77.5	≥ 1.8	Tier 2
Bjørnsen et al., 2019^48^	Powerlifter	17	24.0/26.0	M/F	176.0/177.0	89.0/102	≥ 4	Tier 3
Amani-Shalamzari et al., 2019^27^	Soccer	12	23.0 ± 2.0	M	174.0 ± 5.0	67.5 ± 6.8	≥ 5	Tier 3
Amani-Shalamzari et al., 2020^49^	Soccer	12	23.0 ± 2.0	M	174.0 ± 5.0	67.5 ± 6.8	≥ 5	Tier 3
Elgammal et al., 2020^50^	Basketball	24	22.3 ± 2.4	M	195.4 ± 2.4	81.2 ± 4.7	12	Tier 2
Held et al., 2020^51^	Rower	31	21.9/21.7	M/F	180.4/180.7	73.6/72.5	≥ 8.2	Tier 2
Chen et al., 2022a^52^	Runner	20	21.5/21.6	M	175.0/180.1	66.3/71.7	≥ 6.9	Tier 3
Chen et al., 2022b^53^	Runner	20	21.5/21.6	M	175.0/180.1	66.3/71.7	≥ 6.9	Tier 3
Giovanna et al., 2022^54^	Endurance	19	25.6 ± 5.7	M	176.0 ± 4.4	79.1 ± 15.3	NR	Tier 2
Hosseini Kakhak et al., 2022^25^	Soccer	19	15.9 ± 0.8	M	168.6 ± 7.7	57.6 ± 9.7	≥ 3	Tier 3
Yang et al., 2022^28^	Gymnast	15	13.9 ± 0.4	M/F	156.7/152.4	43.1/43.6	≥ 1	Tier 3
Korkmaz et al., 2022^55^	Soccer	23	18.3/18.4	M	179.0/182.0	71.5/76.0	NR	Tier 2
Wang et al., 2022^56^	Volleyball	12	20.2/20.8	M	184.7/180.0	74.5/69.8	NR	Tier 3
Ugur et al., 2023^57^	Canoe	33	18.6/18.8	M	177.3/177.8	74.7/73.3	≥ 5	Tier 3
Sarfabadi et al., 2023^58^	Long jump	15	NR	NR	162.7/161.6	64.4/65.5	≥ 0.5	Tier 2

N, number of participants; M, male; F, female; TE, training experience; NR, not reported; SFL, sports and fitness levels-classification of athletes into 4 tiers based on training volume and performance metrics (sport level, award winning performance), referenced in McKay et al. study^59^; Tier 2, trained; Tier 3, highly trained.

Furthermore, among the intervention characteristics included in the study, the training duration ranged from 1.1 to 10 weeks, the training frequency ranged from 2 to 14 times per week, and the training protocols were categorized as BFR combined with fixed equipment weights or self-loading, whereas BFR combined with resistance was predominantly low load. Meanwhile, the cuffs were positioned on the proximal thigh or arm, with pressures ranging from 88.2 to 240 mmHg, widths from 3.3 to 14.2 cm, and pressurization times ranging from approximately 5–45 min, mostly with continuous pressurization, as detailed in Table 3.

**Table 3:** Characteristics of BFRT interventions and main outcomes.

References	Design	Intervention characteristics			Comparison	Outcomes
		Duration/frequency	Training protocol/training load	Cuff location/width/pressure/time/pressurization status		
Takarada et al., 2002^38^	RCT	8 weeks, 2 times/week	Knee extension, 4 sets × 15–17 reps/ Moderate	Proximal thighs, 3.3 cm, 200 mmHg, 10 min, Continuous	ML-BFRT (n = 6), ML-RT (n = 6)	Strength (PKE ↑), Body composition (BM ↔ , CSA ↑)
Abe et al., 2005^39^	RCT	8 days, 14 times/week	Squat and leg curl, 3 sets × 15 reps/ Low	Proximal thighs, 3.3 cm, 160–240 mmHg, 6–7 min, Continuous	LL-BFRT (n = 9), LL-RT (n = 6)	Strength (1RM ↑), Speed (10 m ↑, 30 m ↑), Body composition (BM ↔ , CSA ↑, MT ↑, BG ↑)
Sakuraba et al., 2009^40^	RCT	4 weeks, 2 times/week	Knee extension and flexion, 3 sets × 10 reps/Low	Proximal thighs, NR, 200 mmHg, 15 min, NR	LL-BFRT (n = 6), LL-RT (n = 6)	Strength (PKE ↑), Body composition (CSA ↑)
Park et al., 2010^19^	RCT	2 weeks, 12 times/week	Walk, 5 sets × 3 min/ Low	Proximal thighs, 11 cm, 160–220 mmHg, 22 min, Intermittent	LL-BFRT (n = 7), LL-RT (n = 5)	Strength (PKF ↑, PKE ↑), Endurance (VO_2max_ ↑), Body composition (BM ↔)
Yamanaka et al., 2012^9^	RCT	4 weeks, 3 times/week	Bench press and squat, 4 sets × 20–30 reps/ Low	Proximal thighs and proximal arm, 5 cm, Pulled to overlap 2 in., 10 min, Continuous	LL-BFRT (n = 16), LL-RT (n = 16)	Strength (1RM ↑), Body composition (BM ↔ , BG ↑)
Godawa et al., 2012^41^	RCT	10 weeks, 2 times/week	Bench press and squat, 5 sets × 2–5 reps/ High	Proximal knee on the femur, NR, NR, 10–12 min, Continuous	HL-BFRT (n = 8), HL-RT (n = 10)	Strength (1RM ↑), Body composition (BM ↔)
Manimmanakorn et al., 2013a^24^	RCT	5 weeks, 3 times/week	Knee extension and flexion, 3 sets × 22–36 reps/ Low	Proximal thighs, 5 cm, 160–230 mmHg, 12 min, Continuous	LL-BFRT (n = 10), LL-RT (n = 10)	Strength (PKE ↑), Power (CMJ ↑), Speed (10 m ↑),Endurance (VO_2max_ ↑, RP ↑), Body composition (CSA ↑)
Manimmanakorn et al., 2013b^42^	RCT	5 weeks, 3 times/week	Knee extension and flexion, 3 sets × 22–36 reps/ Low	Proximal thighs, 5 cm, 160–230 mmHg, 12 min, Continuous	LL-BFRT (n = 10), LL-RT (n = 10)	Strength (PKE ↑), Body composition (CSA ↑)
Cook et al., 2014^43^	RCT	3 weeks, 3 times/week	Bench press and squat, 5 sets × 5 reps/ High	Proximal thighs, 10.5 cm, 180 mmHg, 20 min, Intermittent	HL-BFRT (n = 10), HL-RT (n = 10)	Strength (1RM ↑), Speed (40 m ↑)
Luebbers et al., 2014^44^	RCT	7 weeks, 4 times/week	Bench press and squat4 sets × 20–30 reps/ Low	Proximal thighs and proximal arm, 7.6 cm, Pulled to overlap 3 in., 10 min, Continuous	LL-BFRT (n = 17), LL-RT (n = 14)	Strength (1RM ↑), Body composition (BG ↑)
Scott et al., 2017^26^	RCT	5 weeks, 3 times/week	Squat, 4 sets × 15–30 reps/ Low	Proximal thighs, 7.5 cm, Perceivedpressure 7/10, 6 min, Continuous	LL-BFRT (n = 10), LL-RT (n = 8)	Power (CMJ ↑), Speed (10 m ↑, 20 m ↑, 40 m ↑),Body composition (MT ↑)
Behringer et al., 2017^45^	RCT	6 weeks, 2 times/week	Sprint, 1 set × 6 reps/ Moderate	Proximal thighs, 13 cm, Pulled to75% length, 8 min, Continuous	ML-BFRT (n = 12), ML-RT (n = 12)	Strength (1RM ↑), Speed (100 m ↑), Body composition (MT ↑)
Amani et al., 2018^46^	RCT	2 weeks, 4 times/week	400 m, 3–4 sets/ Moderate	Proximal thighs, NR, 140–180 mmHg, 8–10 min, NR	ML-BFRT (n = 10), ML-RT (n = 9)	Endurance (VO_2max_ ↑)
Luebbers et al., 2019^47^	RCT	6 weeks, 2 times/week	Back squat, 4 sets × 15–30 reps/ Low	Proximal thighs, 7.6 cm, Pulled to overlap 3 in., 6 min, Continuous	LL-BFRT (n = 8), HL-RT (n = 9)	Strength (1RM ↑)
Bjørnsen et al., 2019^48^	RCT	6.5 weeks, 5 times/week	Front squat, 4 sets × 8–30 reps/ Low	Proximal thighs, 13–14 cm, 120 mmHg, 5 min, Continuous	LL-BFRT (n = 9), HL-RT ( n = 8)	Strength (1RM ↑, PKE ↑), Body composition (CSA ↑, MT ↑)
Amani-Shalamzari et al., 2019^27^	RCT	3 weeks, 3 times/week	SSG, 3 min × 4–8 reps/ High	Proximal thighs, 13 cm, 110–140% SBP, 12–24 min, Intermittent	HL-BFRT (n = 6), HL-RT (n = 6)	Strength (PKF ↑, PKE ↑), Speed (FSP ↑)
Amani-Shalamzari et al., 2020^49^	RCT	3 weeks, 3 times/week	SSG, 3 min × 4–8 reps/ High	Proximal thighs, 13 cm, 110–140% SBP, 12–24 min, Intermittent	HL-BFRT (n = 6), HL-RT (n = 6)	Endurance (VO_2max_ ↑, RP ↑)
Elgammal et al., 2020^50^	RCT	4 weeks, 3 times/week	Sprint, 3 sets × 8 reps/ High	Proximal thighs, 5 cm, 100–160 mmHg, 18–20 min, Intermittent	HL-BFRT (n = 12), HL-RT (n = 12)	Strength (1RM ↑), Speed (143.3 m ↑),Endurance (VO_2max_ ↑)
Held et al., 2020^51^	RCT	5 weeks, 3 times/week	Rowing, 2 sets × 10 min/ Low	Proximal thighs, 13 cm, Pulled to 75% length, 20 min, Continuous	LL-BFRT (n = 16), LL-RT (n = 15)	Strength (1RM ↑)Endurance (VO_2max_ ↑)
Chen et al., 2022a^52^	RCT	8 weeks, 3 times/week	Running, 5 sets × 3 min/ Moderate	Proximal thighs, 14.2 cm, 154 ± 6 mmHg, 20 min, Continuous	ML-BFRT (n = 10), ML-RT (n = 10)	Endurance (RP ↑)
Chen et al., 2022b^53^	RCT	8 weeks, 3 times/week	Running, 5 sets × 3 min/ Moderate	Proximal thighs, 14.2 cm, 153.8 ± 5.7 mmHg, 20 min, Continuous	ML-BFRT (n = 10), ML-RT (n = 10)	Strength (PKF ↑, PKE ↑), Endurance (VO_2max_ ↑, RP ↑)
Giovanna et al., 2022^54^	RCT	2 weeks, 3 times/week	Sprint, 4 sets × 5 reps/High	Proximal thighs, 11 cm, 88.2 ± 10.1 mmHg, 7–10 min, Intermittent	HL-BFRT (n = 10), HL-RT (n = 9)	Endurance (VO_2max_ ↑)
Hosseini Kakhak et al., 2022^25^	RCT	6 weeks, 3 times/week	Soccer drills, SSG, Plyometric, 8–20 min/ Moderate	Proximal thighs, 5 cm, 160–210 mmHg, 45 min, Intermittent	ML-BFRT (n = 10), ML-RT (n = 9)	Strength (1RM ↑), Power (CMJ ↑),Speed (36.3 m ↑), Endurance (RP ↑)
Yang et al., 2022^28^	RCT	10 weeks, 2 times/week	Front and back squat, 3- 4 sets × 10–12 reps/ Low	Proximal thighs, 7.62 cm, Perceived pressure 7/10, 7–10 min, Continuous	LL-BFRT (n = 7), HL-RT ( n = 8)	Power (CMJ ↑),Body composition (BM ↔ , BG↑ )
Korkmaz et al., 2022^55^	RCT	6 weeks, 2 times/week	Leg extension, 4 sets × 15–30 reps/ Low	Proximal thighs, 7 cm, 130–150 mmHg, 6–10 min, Continuous	LL-BFRT (n = 11), HL-RT ( n = 12)	Strength (PKF ↑, PKE ↑),Body composition (MT ↑)
Wang et al., 2022^56^	RCT	8 weeks, 3 times/week	Half squat, 4 sets × 8 reps/ High	Proximal thighs, 7 cm, 180 mmHg, 5 min, Continuous	HL-BFRT (n = 6), HL-RT (n = 6)	Strength (PKF ↑, PKE ↑, 1RM ↑),Power (CMJ ↑)
Ugur et al., 2023^57^	RCT	8 weeks, 2 times/week	Leg press and curl, Extension, 3–4 sets × 10–15 reps/ Low	Proximal thighs, 5 cm, 180–230 mmHg, 15 min, Continuous	LL-BFRT (n = 17), LL-RT (n = 16)	Strength (PKF ↑, PKE ↑),Body composition (CSA ↑, MT ↑)
Sarfabadi et al., 2023 ^58^	RCT	6 weeks, 2 times/week	Leg press, Squat, 3 sets × 15 reps/ Low	Proximal thighs, NR, 150–210 mmHg, 5–10 min, Continuous	LL-BFRT/ML (n = 8) ML-RT (n = 9)	Strength (1RM ↑)

BFRT, blood flow restriction training; RCT, randomized controlled trial; Time, the sum of intermittent pressurization time (minus the intervals) or continuous pressurization time (plus the intervals) during a BFR session; Reps, number of repetition; Training Load, the magnitude of resistance combined with BFR, low (< 50% 1RM or HR_max_ or HR_res_), moderate (50%-70%), high (> 70%); 1RM, 1-repetition maximum; HR_max_, maximal heart rate; HR_res_, heart rate reserve; SSG, small sided game; LL, low load; ML, moderate load; HL, high load; RT, resistance training; NR, not reported; PKF, peak knee flexion; PKE, peak knee extension; BM, body mass; BG, body girths; CSA, muscle cross sectional areas; MT, muscle thickness; VO_2max_, maximal oxygen consumption; CMJ, counter movement jump; RP, running performance; FSP, futsal special performance; ↑, significant within-group improvement from pretest to post-test; ↔ , non-significant within-group change from pretest to post-test.

### Study quality assessment

**Figure 2:**
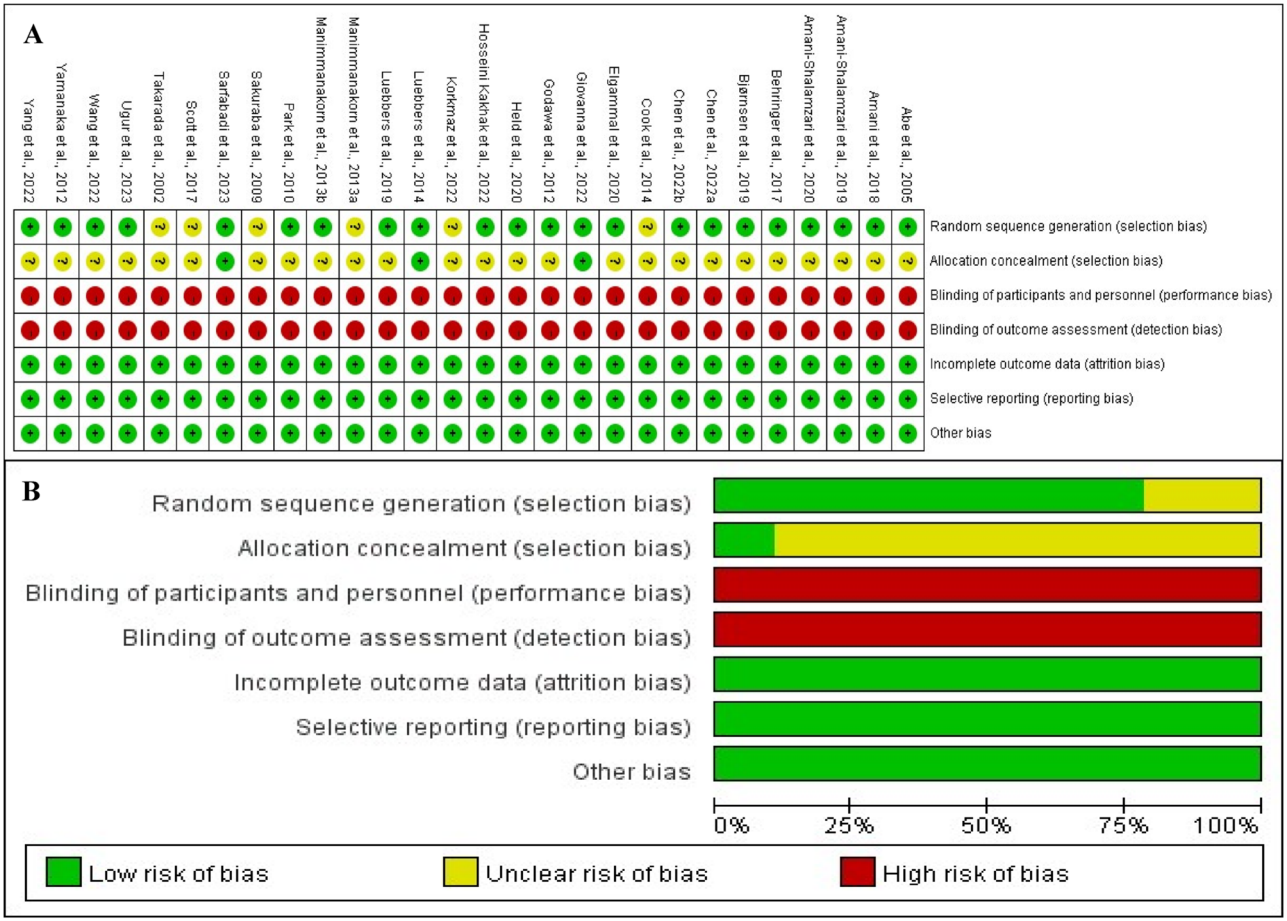
Methodological quality graph and summary of the included studies: (**A**) Risk of bias summary; (**B**) Risk of bias graph.

The overall results of this study after evaluating the methodological quality of 28 RCT articles based on the Cochrane Bias Risk Assessment Tool showed a low risk (see Fig. 2). More specifically, 6 articles did not detail random allocation methods and 25 did not mention allocation concealment, so the selection bias of these articles was categorized as unclear bias. Notably, due to the characteristics of BFRT, athletes could not be blinded to the training intervention, and therefore performance bias was categorized as high risk of bias in all studies. Since high dropout rates were a key factor in missing data and were not reported in all articles, both attrition bias and reporting bias were categorized as low risk of bias.

### *Meta*-analysis results

**Figure 3:**
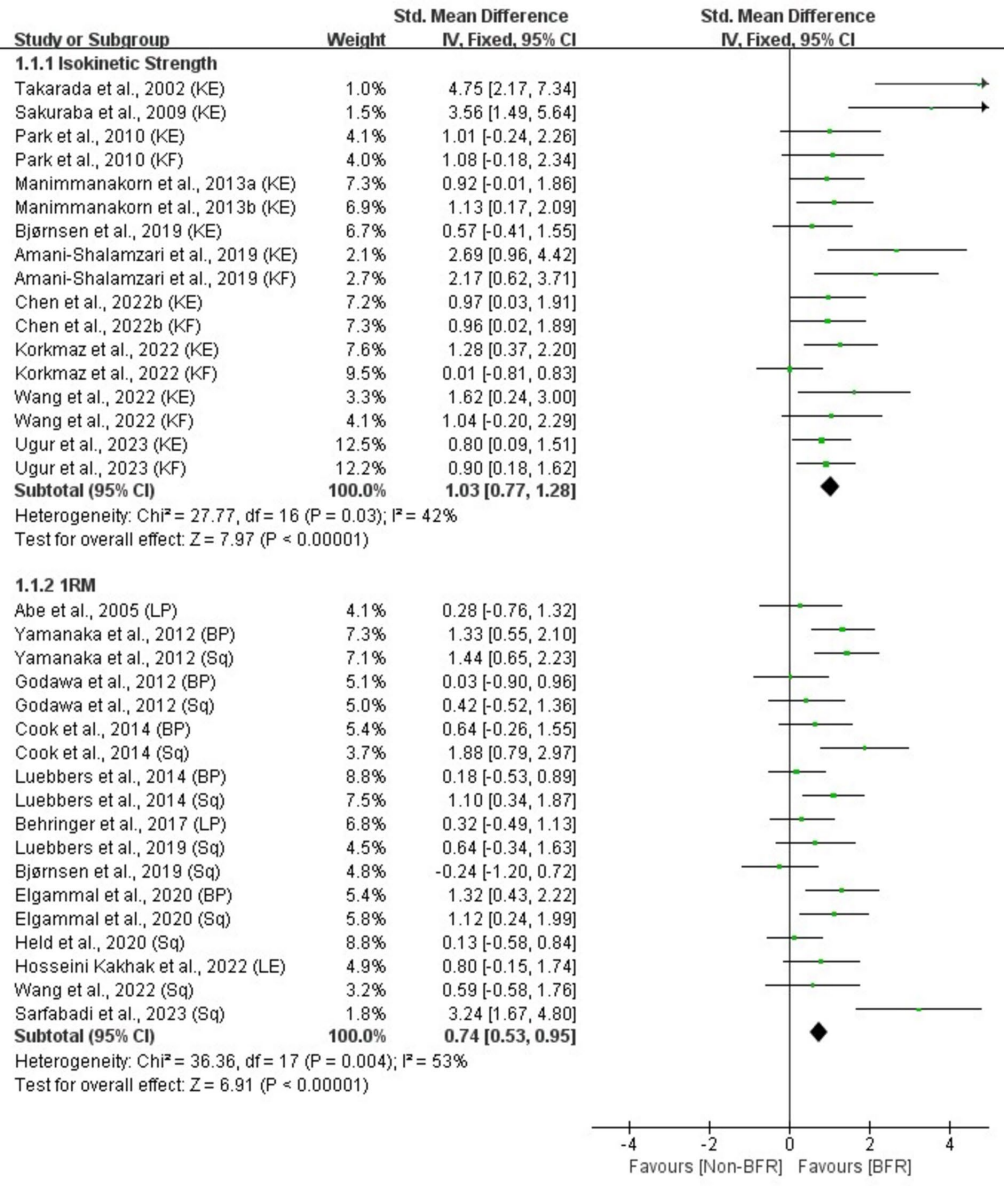
Effect of BFR training versus Non-BFR training on athletes' strength.

**Figure 4:**
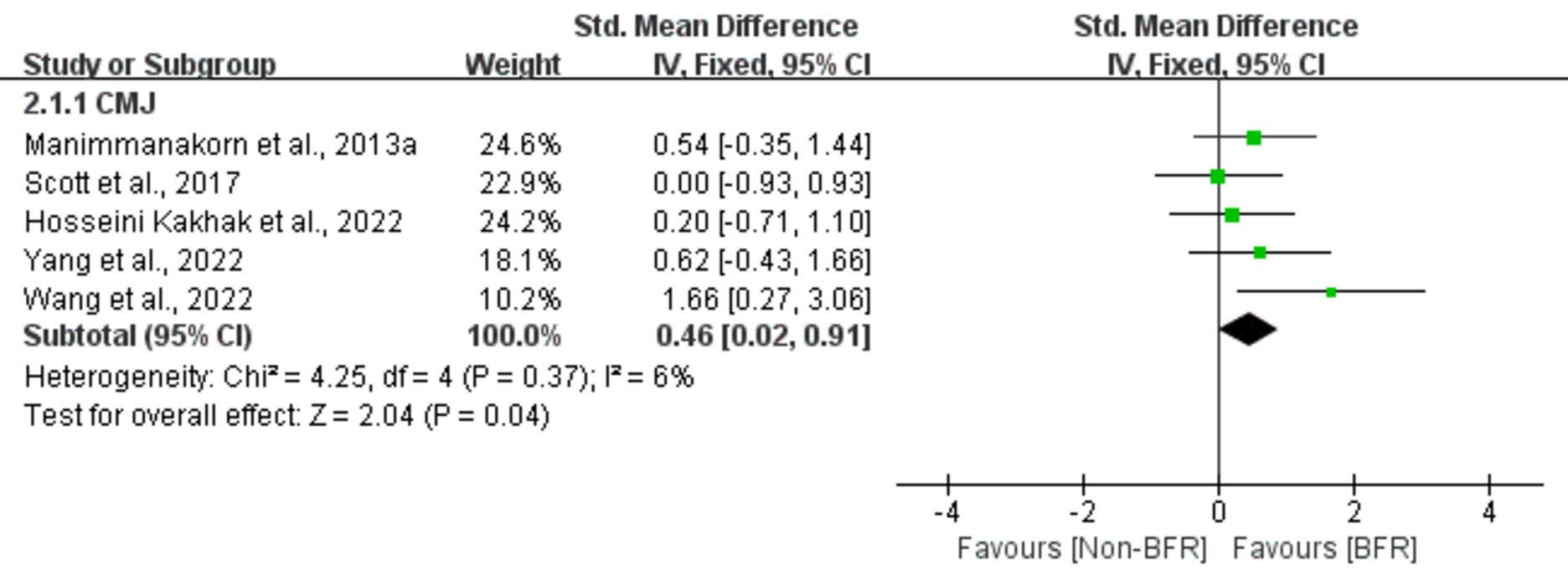
Effect of BFR training versus Non-BFR training on athletes' power.

**Figure 5:**
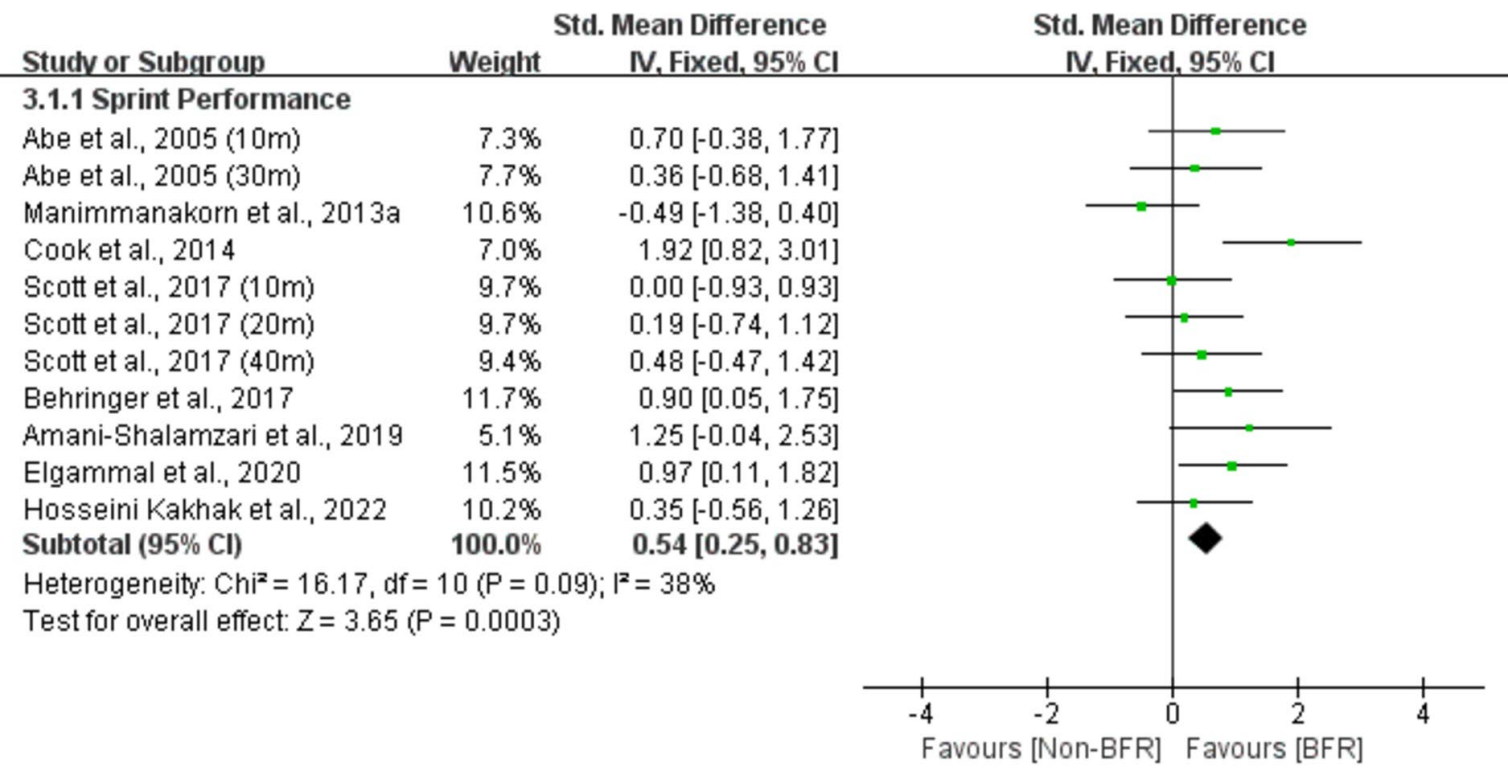
Effect of BFR training versus Non-BFR training on athletes' speed.

**Figure 6:**
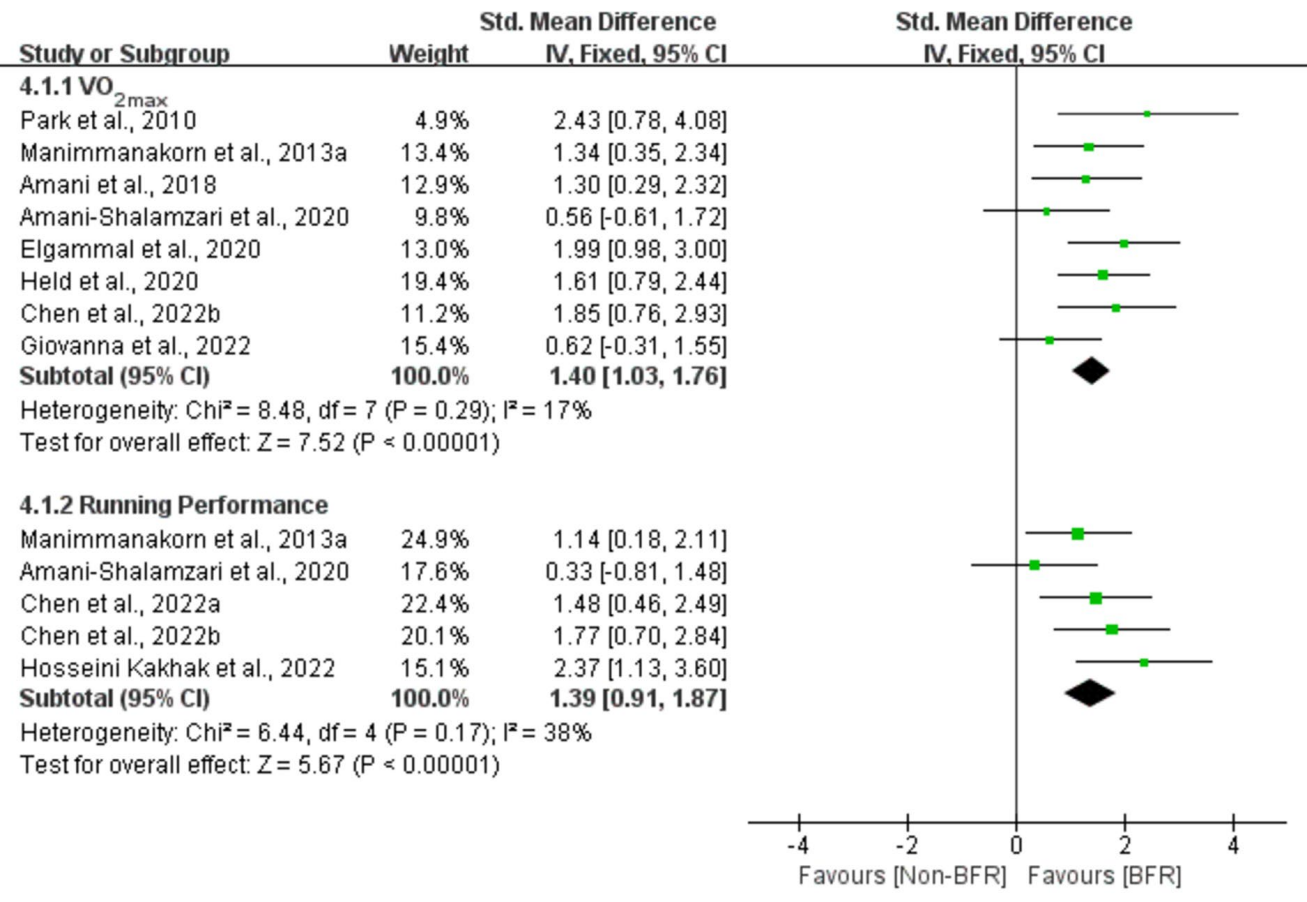
Effect of BFR training versus Non-BFR training on athletes' endurance.

**Figure 7:**
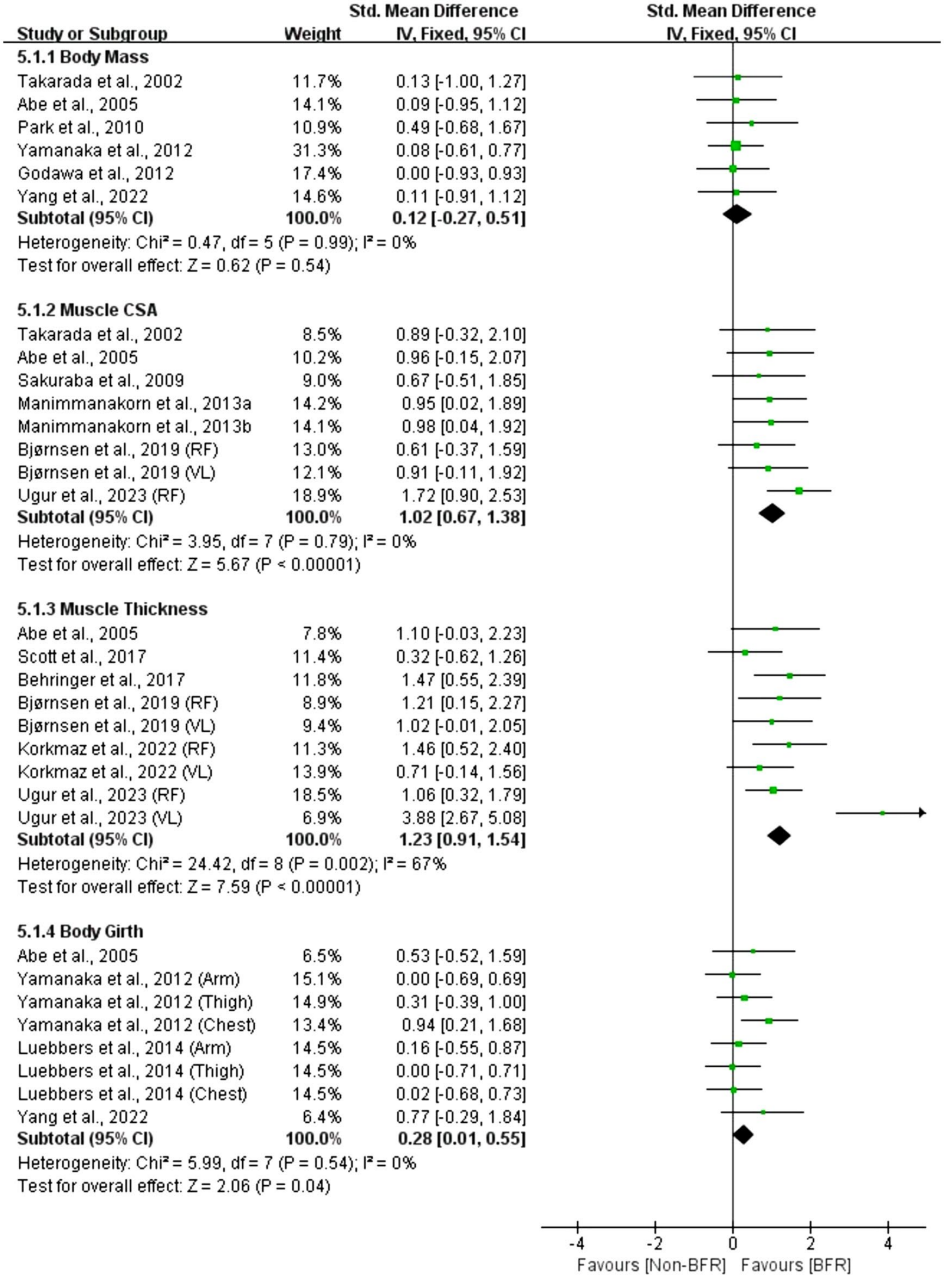
Effect of BFR training versus Non-BFR training on athletes' body composition.

Supplementary Table 1 shows the mean ± SD of physical fitness parameters for the BFR and Non-BFR groups in the included studies. Effect of BFRT on physical fitness parameters: strength (isokinetic strength, 1RM), power (CMJ), speed (sprint performance), endurance (VO_2max_, running performance), body composition (body mass, muscle CSA and thickness, body girth), as shown in Figs. 3, 4, 5, 6 and 7.

### Strength

A meta-analysis of the seventeen included studies showed a moderately significant effect of BFR on isokinetic strength compared to the Non-BFR group (n = 305, SMD = 1.03, 95% CI 0.77–1.28, Z = 7.97, p < 0.001, Fig. 3), with moderate heterogeneity (I^2^ = 42%, p = 0.03). Similarly, a meta-analysis of eighteen included studies showed a moderately significant effect of BFR on 1RM (n = 402, SMD = 0.74, 95% CI 0.53–0.95, Z = 6.91, p < 0.001, Fig. 3) relative to the Non-BFR group, with moderate heterogeneity (I^2^ = 53%, p = 0.004).

### Power

A meta-analysis of the five included studies showed a small significant effect of BFR on CMJ compared to the Non-BFR group (n = 84, SMD = 0.46, 95% CI 0.02–0.91, Z = 2.04, p = 0.04, Fig. 4), and low heterogeneity (I^2^ = 6%, p = 0.37).

### Speed

A meta-analysis of the eleven included studies showed a small significant effect of BFR on sprint performance compared to the Non-BFR group (n = 203, SMD = 0.54, 95% CI 0.25–0.83, Z = 3.65, p < 0.001, Fig. 5), with moderate heterogeneity (I^2^ = 38%, p = 0.09).

### Endurance

A meta-analysis of the eight included studies showed a large significant effect of BFR on VO_2max_ compared to the Non-BFR group (n = 157, SMD = 1.40, 95% CI 1.03–1.76, Z = 7.52, p < 0.001, Fig. 6), with low heterogeneity (I^2^ = 17%, p = 0.29). Similarly, a meta-analysis of five included studies showed a large significant effect of BFR on running performance (n = 91, SMD = 1.39, 95% CI 0.91–1.87, Z = 5.67, p < 0.001, Fig. 6) relative to the Non-BFR group, with moderate heterogeneity (I^2^ = 38%, p = 0.17).

### Body composition

A meta-analysis of the six included studies showed no statistically significant effect of BFR on body mass compared to the Non-BFR group (n = 104, SMD = 0.12, 95% CI − 0.27 to 0.51, Z = 0.62, p = 0.54, Fig. 7), with no heterogeneity (I^2^ = 0%, p = 0.99). But a meta-analysis of the eight included studies showed a moderately significant effect of BFR on muscle CSA compared to the Non-BFR group (n = 146, SMD = 1.02, 95% CI 0.67–1.38, Z = 5.67, p < 0.001, Fig. 7), with no heterogeneity (I^2^ = 0%, p = 0.79). Similarly, a meta-analysis of the nine included studies showed a large significant effect of BFR on muscle thickness (n = 203, SMD = 1.23, 95% CI 0.91–1.54, Z = 7.59, p < 0.001, Fig. 7) relative to the Non-BFR group, with moderate heterogeneity (I^2^ = 67%, p = 0.002). Additionally, a meta-analysis of the eight included studies showed a small significant effect of BFR on body girth compared to the Non-BFR group (n = 219, SMD = 0.28, 95% CI 0.01–0.55, Z = 2.06, p = 0.04, Fig. 7), with no heterogeneity (I^2^ = 0%, p = 0.54).

### Subgroup analyses

A total of 38 subgroup analyses were performed based on the principle of ≥ 3 studies in each moderator, as shown in Supplementary Table 2.

For moderator variables related to training interventions, compared to the Non-BFR group, when training duration ≤ 6 weeks (SMD = 1.09, p < 0.001), frequency ≥ 3 times/week (SMD = 1.11, p < 0.001), high load (SMD = 1.73, p < 0.001), cuff pressure < 160 mmHg (SMD = 1.21, p < 0.001), and pressurization time < 10 min (SMD = 1.32, p < 0.001), the BFR group had a moderate to large significant effect on isokinetic strength. Similarly, when training duration ≤ 6 weeks (SMD = 0.93, p < 0.001), frequency ≥ 3 times/week (SMD = 0.79, p < 0.001), high load (SMD = 0.84, p < 0.001), cuff pressure ≥ 160 mmHg (SMD = 1.00, p < 0.001), the pressurization time ≥ 10 min (SMD = 0.82, p < 0.001), the BFR group had a moderately significant effect on 1RM. Nevertheless, the BFR group had a moderate to large significant effect on CMJ when the training duration > 6 weeks (SMD = 0.99, p = 0.02), high load (SMD = 1.66, p = 0.02), and cuff pressure ≥ 160 mmHg (SMD = 0.59, p = 0.04). Differently, the BFR group had a moderate to large significant effect on sprint performance when training frequency < 3 times/week (SMD = 0.90, p = 0.04), high load (SMD = 1.31, p < 0.001), cuff pressure < 160 mmHg (SMD = 0.60, p = 0.002), and pressurization time ≥ 10 min (SMD = 0.66, p = 0.003).

Furthermore, when the training duration > 6 weeks (SMD = 1.85, p < 0.001), frequency > 3 times/week (SMD = 1.61, p < 0.001), low load (SMD = 1.62, p < 0.001), cuff pressure ≥ 160 mmHg (SMD = 1.59, p < 0.001), the pressurization time ≥ 10 min (SMD = 1.58, p < 0.001), the BFR group had a large significant effect on VO_2max_. Likewise, the BFR group had a large significant effect on running performance when the training duration > 6 weeks (SMD = 1.62, p < 0.001), moderate load (SMD = 1.81, p < 0.001), and cuff pressure ≥ 160 mmHg (SMD = 1.61, p < 0.001). However, when the training duration > 6 weeks (SMD = 1.12, p < 0.001), frequency < 3 times/week (SMD = 1.27, p < 0.001), cuff pressure ≥ 160 mmHg (SMD = 1.11, p < 0.001), and pressurization time ≥ 10 min (SMD = 1.14, p < 0.001) the BFR group had a moderate to large significant effect on muscle CSA. The BFR group had a large significant effect on muscle thickness when the training duration > 6 weeks (SMD = 1.52, p = 0.003), frequency < 3 times/week (SMD = 1.44, p < 0.001), and cuff pressure ≥ 160 mmHg (SMD = 1.65, p < 0.001). In contrast, the BFR group had a small significant effect on body girth when the training duration ≤ 6 weeks (SMD = 0.41, p = 0.03).

### Sensitivity analyses and publication *bias*

Sensitivity analysis showed that no studies leading to highly biased effects were tested by removing them one by one, indicating good robustness of the results, as shown in Supplementary Fig. 1.

Begg's funnel plot was used for assessing publication bias of physical fitness parameters. The funnel plot was shown to be symmetrical with no publication bias for all parameters, as shown in Supplementary Fig. 2.

## Discussion

A meta-analysis of 28 quality assessed low-risk studies was conducted with a total sample size of 542 athletes aged 14–26 years. The results showed that the BFRT intervention had small to large improvements (ES = 0.28–1.40) in athletes' strength (isokinetic strength, 1RM), power (CMJ), speed (sprint performance), endurance (VO_2max_, running performance), body composition (muscle CSA and thickness, body girth), and no improvements in body mass (P > 0.05), compared to training without BFR. However, subgroup analyses demonstrated that training interventions (duration, frequency, load, cuff pressure and pressurization time) also had small to large effects on athletes' physical fitness parameters.

Strength qualities are an important part and the basis of physical fitness parameters, is muscle overcoming external resistance to do work level, and at the same time are closely related to the athletic performance of excellent athletes^60^. The results of the present study indicated that BFRT had a moderately significant effect on isokinetic strength and 1RM compared to Non-BFR training (Non-BFRT) (ES = 0.74–1.03). Similarly, previous meta-analysis found a moderate improvement effect on lower extremity muscle strength levels in healthy individuals (including a small proportion of athletes) after BFRT^61^. With regard to meta-analysis on upper extremity muscle strength, BFR training was more favorable to improve the bench press 1RM in healthy adults compared to Non-BFRT^62^. Indeed, the improvement in strength is due to the hypoxic environment created by BFR, which is more beneficial for muscle protein synthesis, gene expression in myocytes, and muscle fiber recruitment^63,64^. Therefore, BFRT does have a positive effect on the improvement of muscle strength in athletes, and the present meta-analysis supports and reinforces previous findings. Moreover, subgroup analyses showed that athletes with shorter duration, higher frequency, and higher loads had greater improvements in strength quality after BFRT (ES = 1.09–1.73). Meanwhile, when cuff pressure ≥ 160 mmHg and pressurization time ≥ 10 min, the BFR group had a better improvement in 1RM (ES = 0.82–1.00), but the BFR group had a good improvement in isokinetic strength (ES = 1.21–1.32) opposite to the above BFR intervention (cuff pressure < 160 mmHg and pressurization time < 10 min). Likewise, regarding partial BFRT interventions, previous systematic reviews have found results consistent with the current findings^61,65^. From the physiological point of view, high training frequency and high load of BFRT increase the frequency of muscle activation effects and motor units^56^, as well as cuff pressure accelerates metabolic accumulation and hormone secretion levels^66^. More specifically, the BFR situation described above opens up more excitatory signaling pathways, as well as motor neurons recruiting more type II muscle fibers, while promotes more protein synthesis, lactate buildup, and growth hormone release^20,67,68^. However, it is known that the benefits of low loading can reduce the risk of injury and recovery time after training^69^, but the ES values at high loads were greater than at low loads in this study, and the reason for the advantage that high loads have can be explained by the difference in exercise type (e.g. endurance training (walking, sprints) and strength training (bench press, squat)) and athlete type. It is worth noting that high-load strength training can be more beneficial in developing muscular strength in athletes compared to low-load endurance training^12,19,56^. Thus, with regard to strength training protocols for athletes, the above mentioned BFRT intervention protocols can be referred to, taking into account the athlete and exercise type.

Power quality is the production of maximum kinetic energy in a relatively short time, which can also be called explosive power, and also reflects the level of intermuscular coordination and the level of speed of force combination^70,71^. The results of the present study indicated that BFRT had a small significant effect on CMJ compared to Non-BFRT (ES = 0.46). Similarly, previous meta-analysis showed that BFRT better improves lower extremity explosive power (including CMJ performance) in healthy individuals^72^. Actually, the improvement in explosive power is a result of the rapid emergence of neuromuscular adaptive responses in the body in BFR situations, such as the constantly changing number of fast muscle fibers^73^, so BFRT does have an effect on getting improvements in power. Furthermore, the results of the subgroup analysis in this study showed that BFRT with longer duration, high loads and high cuff pressures better developed athletes' explosive power levels (ES = 0.59–1.66). Similarly, Cook et al.^43^ found that when a high load, higher cuff pressure was applied in the BFR group, the CMJ of rugby players improved significantly (1.8% ± 0.7%, p < 0.001). However, Horiuchi et al.^74^ showed no significant improvement in CMJ performance in the BFR group after completing 4 weeks of vertical jumping in healthy young people under high cuff pressure. The explanation for this is that improvements in power are driven by neural mechanisms and are achieved by training with certain loads^75^, so the above may be the result of lower loads failing to achieve improvements in jumping performance. More specifically, high loads during BFRT may have led to more intense neuromuscular adaptations, in which high mechanical stimulus signals promoted a more significant increase in type III and IV afferent neural activity, as well as metabolic compensatory mechanisms that met muscle activation levels more rapidly, resulting in better jumping performance in athletes^56,76^. Therefore, for power training in athletes, BFRT can be referred to the above intervention program, taking into account the type of athlete and exercise.

Speed qualities reflect the level of acceleration and maximal velocity of an athlete during movement, as well as being critical to athletic performance in all sports^77^. Our study findings indicated a small significant improvement in sprint performance with BFRT compared to Non-BFRT (ES = 0.54). Similarly, with regard to the meta-analysis of BFRT for healthy populations, the results found were consistent with the results of this study^72^. Analyzing the physiological mechanisms, BFR creates an environment that better stimulates sympathetic nerve activity thereby increasing reaction speed, as well as stimulating white muscle recruitment resulting in altered movement and displacement speed^78^. Thus, BFRT has a better effect on speed improvement in athletes. Additionally, subgroup analyses indicated that sprint performance improved more significantly in the BFR group when athletes were pressurized with high loads, cuff pressures < 160 mmHg, and ≥ 10 min. Mckee et al.^79^ showed that repetitive sprint training with high loads of BFR had a positive effect on sprint performance in a healthy population. But another previous study found that when the cuff pressure was 150 mmHg, the increase in sprinting ability and acceleration level after LL-BFRT was not statistically different from the Non-BFR group^26^. A better explanation is that although high load mechanical stress leads to increased physical fatigue and oxygen consumption, BFR can improve the energy supply system and accelerate the rate of ATP production and metabolite removal to alleviate fatigue^80^. From a cellular mechanism, BFR in combination with high load may more strongly promotes increased intracellular H + and Pi concentrations, elevated lactate, and decreased PH, while extracellular central nervous system fatigue is rapidly recovered^20,81^. Additionally, different athlete types and exercises were also found to explain the existence of differences based on the intervention characteristics reported in this study, with strength training likely to develop speed aspects superior to endurance training^12,26^. Therefore, when using BFRT to develop an athlete's speed performance, athlete characteristics and exercise type should be considered.

Endurance qualities are the ability of an athlete to maintain high quality movement doing work for a defined period of time and includes aerobic endurance which assesses an athlete's ability to work aerobically^82^. Our results indicated that BFRT had a greater significant effect on VO_2max_ and running performance compared to Non-BFRT (ES = 1.39–1.40). Differently, previous meta-analysis showed that athletes' aerobic capacity was higher than baseline levels after BFRT, but was not statistically different compared to the increase in the Non-BFR group^31^. Nevertheless, another systematic review showed that the use of high-load interval training in the BFR group was effective in improving aerobic capacity in healthy individuals^83^. From a physiological system perspective, both VO_2max_ and running performance of athletes are aerobic endurance parameters in physical cardiovascular endurance, and the increased level of aerobic endurance may be due to the fact that BFR increases oxygen utilization and glycogen synthesis rate while decreasing fatigue accumulation^84^. Thus, the present study supports that BFRT has a better effect on improving endurance qualities in athletes. Additionally, subgroup analyses showed that athletes with longer duration, lower loads (low or moderate), and higher cuff pressures experienced greater improvements in endurance levels after BFRT (ES = 1.59–1.85). Meanwhile, when training frequency was greater than 3 times per week and pressurization duration was ≥ 10 min, the BFR group showed greater significant improvements in VO_2max_ relative to the Non-BFR group (ES = 1.58–1.61). Likewise, previous studies found results consistent with the current findings^85,86^. Interestingly, different cuff pressures cause different changes in the body's internal environment and metabolites, and greater cuff pressures may improve cardiovascular markers^87,88^. Therefore, when athletes develop endurance qualities, coaches can refer to the above BFRT protocol for practice.

Body composition is represented by indicators of substance content and structural proportions inside and outside the human body, which are closely related to metabolic status and bodily functions^89^. Our results showed that BFRT had small to large significant effects (ES = 0.28–1.23) on muscle CSA and thickness, body girth, but not statistically significant (P > 0.05) on body mass compared to Non-BFRT. Nevertheless, a previous meta-analysis found that BFRT not only reduces body mass and body girth in obese populations, but also reduces the risk of cardiovascular disease^90^. Centner et al.^91^ meta-analysis found a greater significant improvement in muscle mass (ES = 1.82) while muscle hypertrophy was less altered (ES = 0.21) in older adults in the BFR group compared to the Non-BFR group, which is consistent with the results of this study. Similarly, one study found that BFRT significantly increased pectoral muscle thickness in older females (p < 0.05)^92^. Conceptually, muscle size includes muscle CSA and thickness, and the former and latter are measured using MRI and ultrasound techniques, respectively^93^. From a physiological viewpoint, changes in muscle size are attributed to myofiber accumulation following activation of the mTOR and calmodulin pathways^94^. Therefore, the findings of this study consolidate previous research as well as confirm the role of BFRT in influencing body composition in athletes. Moreover, subgroup analyses in this study indicated that muscle CSA and thickness were better improved when athletes used longer duration, low frequency, high pressure and longer time. However, previous studies have shown that the low load, higher pressure BFR group obtained a significant increase in muscle mass (3.22%, p < 0.05) after 12 weeks of training^95^. Theoretically, different cuff pressures can cause a state of ischemia and hypoxia in the body, which can benefit muscle protein synthesis and myogenesis inhibitor decline^63,64^. Another meta-analysis result also verified that the LL-BFRT group could achieve the muscle mass growth effect of the traditional HL-RT group regardless of the cuff pressure^13^. Indeed, the physiological mechanisms underlying muscle size alterations are unknown, but post-BFR hormonal changes have been suggested to have a potential role. However, another study has shown that there is no statistical correlation between increased hormones and muscle hypertrophy after resistance exercise^96^, so interpretations regarding muscle size must be made with caution. Thus, when athletes use BFRT to develop body composition, the above protocols must be referred to with caution.

The present meta-analysis also has some limitations. Firstly, only 28 articles were included in this meta-analysis, which may have limited the analysis of data on more comprehensive physical fitness parameters. For example, a meta-analysis could not be performed for agility (505 or COD) because fewer than 3 studies assessed this quality parameter. Secondly, this study did not report a range criterion for cuff pressures for the same sports, which may have resulted in the cuff pressure thresholds in the subgroup analysis results of this study not producing optimal BFR for similar athletes. While exact or correlated values for cuff pressure were indicated in all included studies, there were significant differences in sport type between studies. In addition, physically confrontational and non-confrontational athletes have different quality bases and BFR tolerances, and there was only one on gymnastics and two on track and field in this study, so subgroup analyses were not able to categorize sport types to determine optimal cuff pressures for gymnasts or track and field athletes. Therefore, cuff pressures for BFRT were accurately defined by more subsequent studies of the same or similar sports.

## Conclusion

This meta-analysis confirms the positive effects of BFRT on physical fitness parameters in athletes. More specifically, research evidence indicates that BFRT significantly improved athletes' strength, power, speed, endurance, and body composition, but not body mass parameters. However, since there were less than 3 studies on agility, more studies are needed afterwards to refine the results of the meta-analysis. Subgroup analyses in our study found that the BFRT group was more conducive to improvements in physical fitness parameters when training frequency ≥ 3 times/week, cuff pressure ≥ 160 mmHg, and pressurization time ≥ 10 min. Additionally, subsequent studies should further consider moderators of BFRT (e.g., BFR-related materials, safety) to standardize operation and seek to maximize improvement.

### Supplementary Information

Supplementary Information.

## Data Availability

The original contributions in the study are included in the article/supplementary material, further inquiries can be directed to the corresponding authors.
