# Supplementary material for: Effects of blood flow restriction training on physical fitness among athletes: a systematic review and meta-analysis
Source: Sci Rep. 2024 Jul 18;14:16615. doi: 10.1038/s41598-024-67181-9 (PMC11258269; doi:10.1038/s41598-024-67181-9)
Supplement: Supplementary file 1 — Supplementary Information. [file 41598_2024_67181_MOESM1_ESM.docx]

**Effects of blood flow restriction training on physical fitness among athletes: a systematic review and meta-analysis**

**Kun Yang^1,5^, Chen Soon Chee^1^**^*^**, Johan Abdul Kahar^2^, Tengku Fadilah Tengku Kamalden^3^ , Rui Li^1,5^**^*^**& Shaowen Qian^4^**

^1^Department of Sports Studies, Faculty of Educational Studies, Universiti Putra Malaysia, Selangor, Malaysia,

^2^Department of Orthopedics, Faculty of Medicine and Health Sciences, Universiti Putra Malaysia, Selangor, Malaysia,

^3^National Sports Institute, National Sports Complex, Kuala Lumpur, Malaysia,

^4^Department of Physical Education, Wuhan Sports University, Wuhan, China

^5^These authors contributed equally.

***Correspondence:**

Chee Chen Soon, [cschee@upm.edu.my](mailto:cschee@upm.edu.my)

Rui Li, [gs61877@student.upm.edu.my](mailto:gs64155@student.upm.edu.my)

| **References** | **Parameters** | **BFR training** | | | | **Non-BFR training** | | | |
| --- | --- | --- | --- | --- | --- | --- | --- | --- | --- |
|  |  | **Pre** | **Post** | **n** | **Change** | **Pre** | **Post** | **n** | **Change** |
| Takarada et al., 2002 | PKE-60°/s (Nm**)**  BM (kg)  Muscle CSA (cm^2^) | NR  88.9 ± 10.0  NR | NR  89.1 ± 9.0  NR | 6  6  6 | 14.3 ± 2.0  0.2 ± 5.49  10.7 ± 7.0 | NR  92.4 ± 16.6  NR | NR  91.5 ± 15.9  NR | 6  6  6 | 3.2 ± 2.3  -0.9 ± 9.38  5.35 ± 3.5 |
| Abe et al., 2005 | Leg press 1RM (kg)  10 m (s)  30 m (s)  BM (kg)  Thigh girth (cm)  Muscle CSA (cm^2^)  Muscle thickness (mm) | 208 ± 70  1.95 ± 0.11  4.34 ± 0.14  66.1 ± 4.0  51.8 ± 2.8  190 ± 21  NR | 228 ± 75  1.86 ± 0.08  4.26 ± 0.13  66.5 ± 3.6  52.5 ± 2.7  198 ± 22  NR | 9  9  9  9  9  9  9 | 20.0±34.2  -0.09 ± 0.05  -0.08 ± 0.06  0.4 ± 1.79  0.7 ± 1.29  8.0 ± 10.13  2.95 ± 4.0 | 208 ± 53  1.88 ± 0.12  4.25 ± 0.19  67.6 ± 4.4  53.3 ± 1.9  204 ± 15  NR | 218 ± 62  1.83 ± 0.10  4.20 ± 0.16  67.8 ± 4.9  53.3 ± 2.1  202 ± 17  NR | 6  6  6  6  6  6  6 | 10.0 ± 33.3  -0.05 ± 0.06  -0.05 ± 0.10  0.2 ± 2.68  0.0 ± 1.15  -2.0 ± 9.25  -1.0 ± 2.0 |
| Sakuraba et al., 2009 | PKE-60°/s (Nm**)**  Muscle CSA (cm^2^) | 118.0 ± 0.0  98.5 ± 20.3 | 134.0 ± 0.0  104.0 ± 15.7 | 6  6 | 16.0 ± 4.3  5.5 ± 10.5 | 152.0 ± 0.0  NR | 150.0 ± 0.0  NR | 6  6 | 2.0 ± 2.8  0.1 ± 0.1 |
| Park et al., 2010 | PKE-60°/s (Nm)  PKF-60°/s (Nm)  VO_2max_ (ml/min/kg)  BM (kg) | 235.6 ± 42.6  133.6 ± 24.9  48.9 ± 4.8  83.9 ± 4.4 | 236.4 ± 30.3  153.3 ± 24.1  54.5 ± 4.2  83.8 ± 4.8 | 7  7  7  7 | 0.8 ± 19.76  19.7 ± 13.10  5.6 ± 2.41  -0.1 ± 2.46 | 255.6 ± 25.5  130.0 ± 14.3  47.4 ± 4.5  92.3 ± 9.1 | 236.6 ± 21.7  136.4 ± 11.3  46.8 ± 2.4  90.4 ± 8.0 | 5  5  5  5 | -19.0 ± 15.00  6.4 ± 8.15  -0.6 ± 2.28  -2.2 ± 5.41 |
| Yamanaka et al., 2012 | Bench press 1RM (kg)  Squat 1RM (kg)  BM (kg)  Arm girth (cm)  Thigh girth (cm)  Chest girth (cm) | 128.6 ± 16.5  157.3 ± 20.2  91.3 ± 2.4  35.3 ± 2.8  56.8 ± 4.4  99.8 ± 5.0 | 137.9 ± 17.1  171.4 ± 22.4  91.5 ± 2.4  36.7 ± 2.8  58.0 ± 4.6  102.3 ± 4.4 | 16  16  16  16  16  16 | 9.3 ± 3.4  14.0 ± 3.8  0.2 ± 3.39  1.4 ± 0.98  1.2 ± 1.59  2.6 ± 1.40 | 115.7 ± 14.7  149.4 ± 23.0  89.7 ± 2.9  33.4 ± 2.6  55.6 ± 4.6  99.9 ± 5.3 | 119.8 ± 16.2  156.6 ± 24.3  89.6 ± 2.7  34.8 ± 2.5  56.3 ± 4.5  101.1 ± 5.2 | 16  16  16  16  16  16 | 4.1 ± 4.2  7.2 ± 5.3  -0.1 ± 3.96  1.4 ± 0.90  0.7 ± 1.60  1.2 ± 1.50 |
| Godawa et al., 2012 | Bench press 1RM (kg)  Squat 1RM (kg)  BM (kg) | 147.9 ± 37.6  231.3 ± 50.8  89.97 ± 22.52 | 150.6 ± 34.9  245.8 ± 51.3  88.75 ± 0.0 | 8  8  8 | 2.7 ± 18.1  14.5 ± 25.5  1.21 ± 7.96 | 104.8 ± 43.5  153.8 ± 52.6  77.52 ± 17.79 | 107.0 ± 43.1  157.4 ± 55.3  75.85 ± 0.0 | 10  10  10 | 2.2 ± 19.4  3.6 ± 24.1  1.21 ± 5.62 |
| Manimmanakorn et al., 2013a | PKE-60°/s (Nm)  CMJ (cm)  10 m (s)  VO_2max_ (ml/min/kg)  RP (km/h)  Muscle CSA (cm^2^) | NR  NR  NR  NR  NR  NR | NR  NR  NR  NR  NR  NR | 10  10  10  10  10  10 | 12.1 ± 7.8  7.3 ± 10.6  -0.23 ± 0.05  10.0 ± 3.6  0.6 ± 0.19  6.6 ± 4.5 | NR  NR  NR  NR  NR  NR | NR  NR  NR  NR  NR  NR | 10  10  10  10  10  10 | 1.0 ± 14.3  2.4 ± 6.0  -0.18 ± 0.06  4.2 ± 4.6  0.3 ± 0.3  2.9 ± 2.7 |
| Manimmanakorn et al., 2013b | PKE-60°/s (Nm)  Muscle CSA (cm^2^) | NR  NR | NR  NR | 10  10 | 13.3 ± 6.8  6.6 ± 4.5 | NR  NR | NR  NR | 10  10 | 0.8 ± 13.4  2.9 ± 2.7 |
| Cook et al., 2014 | Bench press 1RM (kg)  Squat 1RM (kg)  40 m (s) | 139.0 ± 7.8  171.5 ± 11.9  5.08 ± 0.18 | NR  NR  NR | 10  10  10 | 5.3 ± 2.2  7.8 ± 2.1  -0.03 ± 0.01 | 141.0 ± 13.6  174.8 ± 13.6  5.11 ± 0.18 | NR  NR  NR | 10  10  10 | 3.3 ± 3.6  4.3 ± 1.4  -0.01 ± 0.01 |
| Luebbers et al., 2014 | Bench press 1RM (kg)  Squat 1RM (kg)  Arm girth (cm)  Thigh girth (cm)  Chest girth (cm) | 123.3 ± 20.7  193.2 ± 25.0  34.7 ± 3.0  59.5 ± 4.7  102.2 ± 10.3 | 132.0 ± 24.4  218.0 ± 24.6  35.3 ± 3.0  61.5 ± 3.9  101.7 ± 9.7 | 17  17  17  17  17 | 8.7 ±7.7  24.9 ± 8.5  0.6 ± 1.02  2.0 ± 1.48  -0.5 ± 3.43 | 135.1 ± 19.7  196.6 ± 27.6  36.3 ± 3.8  61.1 ± 5.1  107.1 ± 13.9 | 142.4 ± 19.0  210.9 ± 26.8  36.7 ± 3.7  63.1 ± 5.9  106.5 ± 11.4 | 14  14  14  14  14 | 7.3 ± 7.3  14.3 ± 10.3  0.4 ± 1.42  2.0 ± 2.08  -0.6 ± 4.80 |
| Scott et al., 2017 | CMJ (cm)  10 m (s)  20 m (s)  40 m (s)  VL Muscle thickness (mm) | 51.0 ± 7.0  1.74 ± 0.07  3.01 ± 0.09  5.37 ± 0.15  28.8 ± 6.9 | 51.0 ± 9.0  1.78 ± 0.04  3.04 ± 0.07  5.41 ± 0.12  30.0 ± 7.7 | 10  10  10  10  10 | 0.0 ± 3.6  0.04 ± 0.02  0.03 ± 0.04  0.04 ± 0.06  1. 2 ± 3.2 | 51.0 ± 3.0  1.73 ± 0.07  3.0 ± 0.13  5.38 ± 0.24  31.9 ± 0.66 | 51.0 ± 3.0  1.77 ± 0.07  3.02 ± 0.11  5.38 ± 0.19  32.0 ± 7.3 | 8  8  8  8  8 | 0.0 ± 1.5  0.04 ± 0.03  0.02 ± 0.06  0.0 ± 0.10  0.1 ± 3.4 |
| Behringer et al., 2017 | Leg press 1RM (kg)  100 m (s)  RF Muscle thickness (mm) | 540 ± 160  12.42 ± 0.71  26.0 ± 3.0 | 600 ± 160  12.05 ± 0.67  28.0 ± 3.0 | 12  12  12 | 50.0 ± 65.3  -0.38 ± 0.28  1.5 ± 1.1 | 430 ± 140  12.39 ± 0.48  25.0 ± 1.0 | 460 ± 130  12.22 ± 0.45  25.0 ± 2.0 | 12  12  12 | 30.0 ± 55.2  -0.16 ± 0.18  0.1 ± 0.7 |
| Amani et al., 2018 | VO_2max_ (ml/min/kg) | 54.6 ± 6.99 | 56.6 ± 7.74 | 10 | 2.0 ± 3.3 | 52.22 ± 5.04 | 50.22 ± 5.4 | 9 | -2.0 ± 2.46 |
| Luebbers et al., 2019 | Squat 1RM (kg) | 88.0 ± 20.6 | 102.3 ± 21.7 | 8 | 14.2 ± 10.1 | 92.7 ± 22.5 | 99.2 ± 20.7 | 9 | 6.6 ± 12.1 |
| Bjørnsen et al., 2019 | Squat 1RM (kg)  PKE-60°/s (Nm)  RF Muscle CSA (cm^2^)  VL Muscle CSA (cm^2^)  RF Muscle thickness (mm)  VL Muscle thickness (mm) | 141.0 ± 25.0  283..0 ± 42  12.1 ± 5.4  23.5 ± 4.5  23.0 ± 4.0  30.0 ± 4.0 | NR  NR  13.0 ± 5.4  25.1 ± 4.5  24.0 ± 4.0  31.3 ± 4.0 | 9  9  9  9  9  9 | 4.1 ± 7.27  9.4 ± 14.3  0.97 ± 1.47  1.64 ± 1.88  1.1 ± 0.6  1.3 ± 1.1 | 151.0 ± 26.0  315.0 ± 68  14.8 ± 4.4  24.0 ± 3.2  24.0 ± 4.0  34.0 ± 4.0 | NR  NR  15.0 ± 4.4  24.1 ± 3.2  23.6 ± 4.0  33.6 ± 4.0 | 8  8  8  8  8  8 | 5.9 ± 6.85  -1.8 ± 22.6  0.21 ± 0.73  0.12 ± 1.17  -0.4 ± 1.6  -0.4 ± 2.0 |
| Amani-Shalamzari et al., 2019 | PKE-60°/s(Nm)  PKF-60°/s (Nm)  FSP (s) | 185.8 ± 22.0  83.2 ± 13.1  34.9 ± 2.5 | 242.0 ± 12.7  102.3 ± 9.6  30.4 ± 2.6 | 6  6  6 | 56.2 ± 10.4  19.1 ± 6.6  -4.5 ± 1.9 | 190.9 ± 20.6  81.4 ± 8.3  33.8 ± 3.1 | 218.5 ± 9.2  87.7 ± 5.3  31.8 ± 3.2 | 6  6  6 | 27.6 ± 9.2  6.3 ± 4.0  -2.0 ± 1.8 |
| Amani-Shalamzari et al., 2020 | VO_2max_ (ml/min/kg)  RP (km/h) | 44.0 ± 5.7  15.8 ± 0.9 | 48.7 ± 5.3  16.4 ± 1.1 | 6  6 | 4.7 ± 3.17  0.6 ± 0.6 | 37.1 ± 5.2  14.9 ± 0.7 | 39.8 ± 6.6  15.3 ± 0.9 | 6  6 | 2.7 ± 3.43  0.4 ± 0.5 |
| Elgammal et al., 2020 | Bench press 1RM (kg)  Squat 1RM (kg)  143.3 m (s)  VO_2max_ (ml/min/kg) | 76.5 ± 6.0  127.5 ± 10.1  32.3 ± 0.7  38.3 ± 2.2 | 87.3 ± 6.9  150.1 ± 10.6  32.1 ± 0.8  46.2 ± 3.1 | 12  12  12  12 | 10.8 ± 2.6  22.7 ± 8.2  -0.3 ± 0.1  7.9 ± 1.09 | 74.8 ± 5.5  126.0 ± 7.3  32.5 ± 0.6  38.1 ± 1.5 | 82.1 ± 6.6  140.4 ± 7.5  32.3 ± 0.6  43.9 ± 2.9 | 12  12  12  12 | 7.3 ± 2.5  14.4 ± 6.0  -0.2 ± 0.1  5.8 ± 0.94 |
| Held et al., 2020 | Squat 1RM (kg)  VO_2max_ (ml/min/kg) | 106.2 ± 20.0  63.0 ± 7.0 | 111.9 ± 20.9  69.7 ± 9.4 | 16  16 | 5.7 ± 7.2  6.7 ± 2.93 | 99.1 ± 25.1  63.2 ± 8.5 | 103.7 ± 25.4  64.9 ± 8.6 | 15  15 | 4.6 ± 9.2  1.7 ± 3.12 |
| Chen et al., 2022a | RP (km/h) | 13.52 ± 1.87 | 15.15 ± 1.08 | 10 | 1.63 ± 0.68 | 13.00 ± 1.56 | 13.59 ± 1.43 | 10 | 0.59 ± 0.67 |
| Chen et al., 2022b | PKE-60°/s (Nm)  PKF-60°/s (Nm)  VO_2max_ (ml/min/kg)  RP (km/h) | 150.5 ± 8.6  89.5 ± 6.6  64.25 ± 1.49  9.81 ± 0.25 | 173.1 ± 9.3  102.1 ± 5.3  66.01 ± 0.93  11.25 ± 0.37 | 10  10  10  10 | 22.6 ± 16.5  12.6 ± 9.1  1.76 ± 0.56  1.44 ± 0.14 | 179.8 ± 6.4  105.3 ± 8.6  60.32 ± 1.71  9.56 ± 0.29 | 171.2 ± 5.7  107.5 ± 5.7  60.94 ± 0.93  10.75 ± 0.29 | 10  10  10  10 | 8.6 ± 10.4  2.2 ± 11.6  0.62 ± 0.62  1.19 ± 0.13 |
| Giovanna et al., 2022 | VO_2max_ (ml/min/kg) | 51.9 ± 4.6 | 54.8 ± 7.7 | 10 | 2.9 ± 2.83 | 54.0 ± 6.9 | 54.9 ± 7.3 | 9 | 0.9 ± 3.34 |
| Hosseini Kakhak et al., 2022 | Leg extension 1RM (kg)  CMJ (cm)  36.3 m (s)  RP (km/h) | 68.0 ± 7.9  38.1 ± 4.60  5.9 ± 0.4  6.73 ± 1.0 | 82.5 ± 6.8  42.5 ± 3.5  5.5 ± 0.3  10.5 ± 0.86 | 10  10  10  10 | 14.5 ± 5.0  4.4 ± 4.4  -0.4 ± 0.24  3.78 ± 0.89 | 74.4 ± 10.1  37.6 ± 7.9  5.9 ± 0.5  7.16 ± 0.70 | 84.4 ± 8.8  41.1 ± 8.8  5.6 ± 0.3  8.7 ± 1.0 | 9  9  9  9 | 10.0 ± 5.8  3.6 ± 3.23  -0.3 ± 0.31  1.61 ± 0.86 |
| Yang et al., 2022 | CMJ (cm)  BM (kg)  Thigh girth (cm) | NR  43.0 ± 7.0  43.0 ± 8.0 | NR  44.4 ± 7.0  45.0 ± 4.3 | 7  7  7 | 5.0 ± 2.6  1.4 ± 0.88  2.0 ± 1.01 | NR  43.0 ± 7.0  44.0 ± 4.0 | NR  44.3 ± 7.0  45.0 ± 4.0 | 8  8  8 | 3.3 ± 2.6  1.3 ± 0.87  1.2 ± 0.94 |
| Korkmaz et al., 2022 | PKE-60°/s (Nm)  PKF-60°/s (Nm)  RF Muscle thickness (mm)  VL Muscle thickness (mm) | 157.4 ± 33.6  108.8 ± 26.6  23.5 ± 3.0  25.1 ± 2.8 | 173.9 ± 27.9  127.1 ± 22.8  26.9 ± 3.9  27.3 ± 4.3 | 11  11  11  11 | 16.5 ± 13.16  18.3 ± 10.6  3.4 ± 1.4  2.2 ± 1.5 | 181.1 ± 22.8  97.4 ± 19.1  25.2 ± 3.5  26.7 ± 4.1 | 182.6 ± 22.3  115.6 ± 19.8  26.4 ± 3.9  27.8 ± 3.3 | 12  12  12  12 | 1.5 ± 9.2  18.2 ± 7.9  1.2 ± 1.5  1.1 ± 1.5 |
| Wang et al., 2022 | PKE-60°/s (Nm)  PKF-60°/s (Nm)  Squat 1RM (kg)  CMJ (cm) | 213.1 ± 11.5  133.9 ± 6.1  190.2 ± 46.3  57.90 ± 8.34 | 250.8 ± 13.5  155.2 ± 7.1  244.6 ± 59.6  NR | 6  6  6  6 | 37.7 ± 7.2  21.3 ± 3.8  54.3 ± 30.8  7.1 ± 4.2 | 208.8 ± 9.6  131.7 ± 6.1  196.7 ± 52.7  59.18 ± 3.72 | 235.7 ± 7.5  148.7 ± 6.9  222.1 ± 59.5  NR | 6  6  6  6 | 26.9 ± 4.9  17.0 ± 3.8  34.0 ± 32.5  1.28 ± 1.8 |
| Ugur et al., 2023 | PKE-60°/s (Nm)  PKF-60°/s (Nm)  RF Muscle CSA (cm^2^)  RF Muscle thickness (mm)  VL Muscle thickness (mm) | 198.2 ± 36.6  110.9 ± 46.1  9.5 ± 2.4  17.4 ± 2.4  19.2 ± 2.8 | 227.1 ± 46.9  132.5 ± 31.0  11.4 ± 2.9  19.1 ± 2.2  23.6 ± 4.3 | 17  17  17  17  17 | 28.9 ± 14.4  21.6 ± 13.5  1.9 ± 0.9  1.7 ± 0.8  4.4 ± 1.2 | 205.3 ± 52.8  108.4 ± 58.3  11.1 ± 2.1  18.6 ± 3.2  21.5 ± 2.4 | 220.1 ± 58.8  116.3 ± 27.4  11.5 ± 2.2  19.2 ± 3.4  21.5 ± 3.2 | 16  16  16  16  16 | 14.8 ± 19.8  7.9 ± 16.1  0.4 ± 0.8  0.6 ± 1.2  0.0 ± 1.0 |
| Sarfabadi et al., 2023 | Squat 1RM (kg) | 80.0 ± 17.3 | 94.4 ± 17.0 | 8 | 14.4 ± 8.6 | 70.4 ± 17.5 | 59.8 ± 4.6 | 9 | -10.6 ± 6.0 |

**Supplementary Table 1.** The mean ± SD physical fitness parameters reported for the BFR training and Non-BFR training in the included studies. BFR, blood flow restriction; PKF, peak knee flexion; PKE, peak knee extension; 1RM, 1-repetition maximum; BM, body mass; CSA, cross sectional areas; RF, rectus femoris; VL, vastus lateralis; VO_2max_, maximal oxygen consumption; CMJ, counter movement jump; RP, running performance; FSP, futsal special performance; NR, not reported.

| **Subgroup** | **N** | **SMD (95%CI)** | **Z** | **P** | **Weight (%)** | **Between Group** | | **Within Group** | |
| --- | --- | --- | --- | --- | --- | --- | --- | --- | --- |
|  |  |  |  |  |  | **I^2^ (%)** | **p** | **I^2^ (%)** | **p** |
| **Isokinetic Strength** | | | | | | | | | |
| ≤ 6 weeks | 9/9/9/146 | 1.09 (0.71, 1.46) | 5.70 | < 0.001 | 45.7 | 0 | 0.67 | 54 | 0.02 |
| > 6 weeks | 8/8/8/159 | 0.98 (0.63, 1.32) | 5.59 | < 0.001 | 54.3 |  |  | 30 | 0.19 |
| < 3 times/week | 6/6/6/136 | 0.92 (0.54, 1.30) | 4.75 | < 0.001 | 44.3 | 0 | 0.46 | 75 | 0.001 |
| ≥ 3 times/week | 11/11/11/169 | 1.11 (0.77, 1.45) | 6.45 | < 0.001 | 55.7 |  |  | 0 | 0.72 |
| Low | 10/10/10/205 | 0.87 (0.57, 1.17) | 5.75 | < 0.001 | 72.3 | 59.8 | 0.08 | 27 | 0.19 |
| Moderate | 3/3/3/52 | 1.20 (0.56, 1.84) | 3.66 | < 0.001 | 15.4 |  |  | 74 | 0.02 |
| High | 4/4/4/48 | 1.73 (1.01, 2.45) | 4.70 | < 0.001 | 12.2 |  |  | 0 | 0.44 |
| < 160 mmHg | 10/10/10/152 | 1.21 (0.84, 1.58) | 6.42 | < 0.001 | 46.7 | 43.7 | 0.18 | 34 | 0.14 |
| ≥ 160 mmHg | 7/7/7/153 | 0.87 (0.52, 1.21) | 4.91 | < 0.001 | 53.3 |  |  | 51 | 0.05 |
| < 10 min | 10/10/10/152 | 1.32 (0.94, 1.70) | 6.82 | < 0.001 | 44.0 | 76.0 | 0.04 | 48 | 0.05 |
| ≥ 10 min | 7/7/7/153 | 0.79 (0.46, 1.13) | 4.61 | < 0.001 | 56.0 |  |  | 7 | 0.38 |
| **1RM** | | | | | | | | | |
| ≤ 6 weeks | 12/12/12/275 | 0.93 (0.67, 1.19) | 7.04 | < 0.001 | 65.7 | 83.6 | 0.01 | 55 | 0.01 |
| > 6 weeks | 6/6/6/127 | 0.37 (0.02, 0.73) | 2.04 | 0.04 | 34.3 |  |  | 17 | 0.30 |
| < 3 times/week | 5/5/5/94 | 0.57 (0.13, 1.00) | 2.55 | 0.01 | 23.2 | 0 | 0.38 | 69 | 0.01 |
| ≥ 3 times/week | 13/13/13/308 | 0.79 (0.55, 1.03) | 6.48 | < 0.001 | 76.8 |  |  | 47 | 0.03 |
| Low | 9/9/9/223 | 0.72 (0.44, 1.01) | 5.00 | < 0.001 | 54.7 | 0 | 0.67 | 69 | 0.001 |
| Moderate | 2/2/2/43 | 0.52 (-0.09, 1.13) | 1.66 | 0.10 | 11.7 |  |  | 0 | 0.45 |
| High | 7/7/7/136 | 0.84 (0.48, 1.20) | 4.55 | < 0.001 | 33.6 |  |  | 34 | 0.17 |
| < 160 mmHg | 12/12/12/299 | 0.66 (0.42, 0.90) | 5.40 | < 0.001 | 76.9 | 45.6 | 0.18 | 48 | 0.03 |
| ≥ 160 mmHg | 6/6/6/103 | 1.00 (0.57, 1.44) | 4.51 | < 0.001 | 23.1 |  |  | 63 | 0.02 |
| < 10 min | 6/6/6/102 | 0.51 (0.09, 0.92) | 2.38 | 0.02 | 25.2 | 36.9 | 0.21 | 66 | 0.01 |
| ≥ 10 min | 12/12/12/300 | 0.82 (0.57, 1.06) | 6.60 | < 0.001 | 74.8 |  |  | 46 | 0.04 |
| **CMJ** | | | | | | | | | |
| ≤ 6 weeks | 3/3/3/57 | 0.25 (-0.27, 0.78) | 0.95 | 0.34 | 71.7 | 53.6 | 0.14 | 0 | 0.70 |
| > 6 weeks | 2/2/2/27 | 0.99 (0.16, 1.83) | 2.33 | 0.02 | 28.3 |  |  | 28 | 0.24 |
| < 3 times/week | 1/1/1/15 | 0.62 (-0.43, 1.66) | 1.15 | 0.25 | 18.1 | 0 | 0.75 | \ | \ |
| ≥ 3 times/week | 4/4/4/69 | 0.43 (-0.06, 0.92) | 1.71 | 0.09 | 81.9 |  |  | 28 | 0.25 |
| Low | 3/3/3/53 | 0.37 (-0.18, 0.92) | 1.33 | 0.18 | 65.6 | 39.1 | 0.19 | 0 | 0.62 |
| Moderate | 1/1/1/19 | 0.20 (-0.71, 1.10) | 0.43 | 0.67 | 24.2 |  |  | \ | \ |
| High | 1/1/1/12 | 1.66 (0.27, 3.06) | 2.34 | 0.02 | 10.2 |  |  | \ | \ |
| < 160 mmHg | 2/2/2/33 | 0.27 (-0.42, 0.97) | 0.77 | 0.44 | 41.0 | 0 | 0.48 | 0 | 0.39 |
| ≥ 160 mmHg | 3/3/3/51 | 0.59 (0.02, 1.17) | 2.01 | 0.04 | 59.0 |  |  | 34 | 0.22 |
| < 10 min | 3/3/3/45 | 0.55 (-0.07, 1.17) | 1.73 | 0.08 | 51.2 | 0 | 0.70 | 48 | 0.15 |
| ≥ 10 min | 2/2/2/39 | 0.37 (-0.26, 1.01) | 1.15 | 0.25 | 48.8 |  |  | 0 | 0.59 |
| **Sprint Performance** | | | | | | | | | |
| < 3 times/week | 1/1/1/24 | 0.90 (0.05, 1.75) | 2.09 | 0.04 | 11.7 | 0 | 0.37 | \ | \ |
| ≥ 3 times/week | 10/10/10/179 | 0.49 (0.18, 0.80) | 3.12 | 0.002 | 88.3 |  |  | 41 | 0.08 |
| Low | 6/6/6/104 | 0.17 (-0.23, 0.56) | 0.83 | 0.41 | 54.5 | 79.8 | 0.007 | 0 | 0.60 |
| Moderate | 2/2/2/43 | 0.64 (0.02, 1.26) | 2.04 | 0.04 | 21.9 |  |  | 0 | 0.38 |
| High | 3/3/3/56 | 1.31 (0.71, 1.90) | 4.29 | < 0.001 | 23.6 |  |  | 0 | 0.41 |
| < 160 mmHg | 6/6/6/114 | 0.60 (0.22, 0.99) | 3.07 | 0.002 | 57.2 | 0 | 0.63 | 0 | 0.47 |
| ≥ 160 mmHg | 5/5/5/89 | 0.46 (0.01, 0.90) | 2.03 | 0.04 | 42.8 |  |  | 65 | 0.02 |
| < 10 min | 6/6/6/108 | 0.45 (0.06, 0.84) | 2.25 | 0.02 | 55.6 | 0 | 0.48 | 0 | 0.77 |
| ≥ 10 min | 5/5/5/95 | 0.66 (0.22, 1.09) | 2.96 | 0.003 | 44.4 |  |  | 70 | 0.01 |
| **VO_2max_** | | | | | | | | | |
| ≤ 6 weeks | 7/7/7/137 | 1.34 (0.95, 1.72) | 6.80 | < 0.001 | 88.8 | 0 | 0.39 | 22 | 0.26 |
| > 6 weeks | 1/1/1/20 | 1.85 (0.76, 2.93) | 3.34 | < 0.001 | 11.2 |  |  | \ | \ |
| ≤ 3 times/week | 6/6/6/126 | 1.35 (0.95, 1.75) | 6.60 | < 0.001 | 82.3 | 0 | 0.59 | 28 | 0.23 |
| > 3 times/week | 2/2/2/31 | 1.61 (0.75, 2.47) | 3.65 | < 0.001 | 17.7 |  |  | 23 | 0.25 |
| Low | 3/3/3/63 | 1.62 (1.03, 2.21) | 5.37 | < 0.001 | 37.8 | 0 | 0.39 | 0 | 0.55 |
| Moderate | 2/2/2/39 | 1.56 (0.82, 2.30) | 4.12 | < 0.001 | 24.1 |  |  | 0 | 0.47 |
| High | 3/3/3/55 | 1.07 (0.48, 1.66) | 3.56 | < 0.001 | 38.1 |  |  | 59 | 0.09 |
| < 160 mmHg | 4/4/4/86 | 1.25 (0.77, 1.73) | 5.12 | < 0.001 | 57.6 | 0 | 0.37 | 49 | 0.11 |
| ≥ 160 mmHg | 4/4/4/71 | 1.59 (1.03, 2.15) | 5.58 | < 0.001 | 42.4 |  |  | 0 | 0.63 |
| < 10 min | 2/2/2/38 | 0.93 (0.25, 1.62) | 2.67 | 0.008 | 28.3 | 59.6 | 0.12 | 0 | 0.33 |
| ≥ 10 min | 6/6/6/119 | 1.58 (1.15, 2.01) | 7.21 | < 0.001 | 71.7 |  |  | 1 | 0.41 |
| **Running Performance** | | | | | | | | | |
| ≤ 6 weeks | 3/3/3/51 | 1.22 (0.58, 1.85) | 3.77 | < 0.001 | 57.5 | 0 | 0.42 | 65 | 0.06 |
| > 6 weeks | 2/2/2/40 | 1.62 (0.88, 2.35) | 4.30 | < 0.001 | 42.5 |  |  | 0 | 0.69 |
| Low | 1/1/1/20 | 1.14 (0.18, 2.11) | 2.33 | 0.02 | 24.9 | 61.9 | 0.07 | \ | \ |
| Moderate | 3/3/3/59 | 1.81 (1.18, 2.44) | 5.62 | < 0.001 | 57.5 |  |  | 0 | 0.55 |
| High | 1/1/1/12 | 0.33 (-0.81, 1.48) | 0.57 | 0.57 | 17.6 |  |  | \ | \ |
| < 160 mmHg | 1/1/1/12 | 0.33 (-0.81, 1.48) | 0.57 | 0.57 | 17.6 | 74.7 | 0.05 | \ | \ |
| ≥ 160 mmHg | 4/4/4/79 | 1.61 (1.08, 2.14) | 5.98 | < 0.001 | 82.4 |  |  | 0 | 0.48 |
| **Muscle CSA** | | | | | | | | | |
| ≤ 6 weeks | 4/4/4/67 | 0.91 (0.40, 1.42) | 3.48 | < 0.001 | 47.5 | 0 | 0.56 | 0 | 0.98 |
| > 6 weeks | 4/4/4/79 | 1.12 (0.63, 1.61) | 4.51 | < 0.001 | 52.5 |  |  | 12 | 0.33 |
| < 3 times/week | 3/3/3/57 | 1.27 (0.68, 1.85) | 4.23 | < 0.001 | 36.4 | 4.2 | 0.31 | 21 | 0.28 |
| ≥ 3 times/week | 5/5/5/89 | 0.88 (0.44, 1.33) | 3.91 | < 0.001 | 63.6 |  |  | 0 | 0.98 |
| < 160 mmHg | 2/2/2/34 | 0.75 (0.05, 1.46) | 2.10 | 0.04 | 25.1 | 0 | 0.39 | 0 | 0.68 |
| ≥ 160 mmHg | 6/6/6/112 | 1.11 (0.70, 1.52) | 5.34 | < 0.001 | 74.9 |  |  | 0 | 0.69 |
| < 10 min | 3/3/3/49 | 0.81 (0.22, 1.41) | 2.68 | 0.007 | 35.3 | 0 | 0.39 | 0 | 0.87 |
| ≥ 10 min | 5/5/5/97 | 1.14 (0.70, 1.57) | 5.07 | < 0.001 | 64.7 |  |  | 0 | 0.57 |
| **Muscle Thickness** | | | | | | | | | |
| ≤ 6 weeks | 5/5/5/103 | 0.99 (0.57, 1.42) | 4.62 | < 0.001 | 56.3 | 62.1 | 0.10 | 10 | 0.35 |
| > 6 weeks | 4/4/4/100 | 1.52 (1.04, 2.00) | 6.24 | < 0.001 | 43.7 |  |  | 83 | < 0.001 |
| < 3 times/week | 5/5/5/136 | 1.44 (1.04, 1.84) | 7.05 | < 0.001 | 62.4 | 65.9 | 0.09 | 80 | < 0.001 |
| ≥ 3 times/week | 4/4/4/67 | 0.87 (0.35, 1.38) | 3.30 | 0.001 | 37.6 |  |  | 0 | 0.58 |
| < 160 mmHg | 6/6/6/122 | 1.01 (0.63 1.40) | 5.13 | < 0.001 | 66.8 | 71.1 | 0.06 | 0 | 0.47 |
| ≥ 160 mmHg | 3/3/3/81 | 1.65 (1.10, 2.20) | 5.89 | < 0.001 | 33.2 |  |  | 88 | < 0.001 |
| **Body Girths** | | | | | | | | | |
| ≤ 6 weeks | 4/4/4/111 | 0.41 (0.03, 0.79) | 2.12 | 0.03 | 50.0 | 0 | 0.34 | 14 | 0.32 |
| > 6 weeks | 4/4/4/108 | 0.15 (-0.23, 0.53) | 0.78 | 0.43 | 50.0 |  |  | 0 | 0.66 |
| < 3 times/week | 1/1/1/15 | 0.77 (-0.29, 1.84) | 1.43 | 0.15 | 6.4 | 0 | 0.35 | \ | \ |
| ≥ 3 times/week | 7/7/7/204 | 0.25 (-0.03, 0.53) | 1.75 | 0.08 | 93.6 |  |  | 0 | 0.53 |
| < 160 mmHg | 7/7/7/204 | 0.27 (-0.01, 0.54) | 1.87 | 0.06 | 93.5 | 0 | 0.63 | 0 | 0.45 |
| ≥ 160 mmHg | 1/1/1/15 | 0.53 (-0.52, 1.59) | 0.99 | 0.32 | 6.5 |  |  | \ | \ |
| < 10 min | 2/2/2/30 | 0.65 (-0.10, 1.40) | 1.70 | 0.09 | 12.9 | 6.4 | 0.30 | 0 | 0.75 |
| ≥ 10 min | 6/6/6/189 | 0.23 (-0.06, 0.52) | 1.55 | 0.12 | 87.1 |  |  | 0 | 0.44 |

**Supplementary Table 2.** Effect of moderator variables with 95% confidence intervals. N, data denote the number of studies providing data for analysis, the number of BFR groups, the number of Non-BFR groups and the total number of athletes included in the analysis, respectively; 1RM, 1-repetition maximum; CMJ, counter movement jump; VO_2max_, maximal oxygen consumption; CSA, cross sectional areas.

**Isokinetic Strength 1RM CMJ**


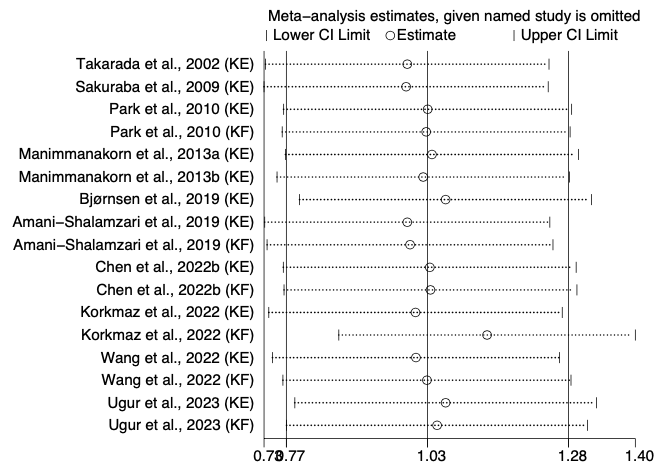

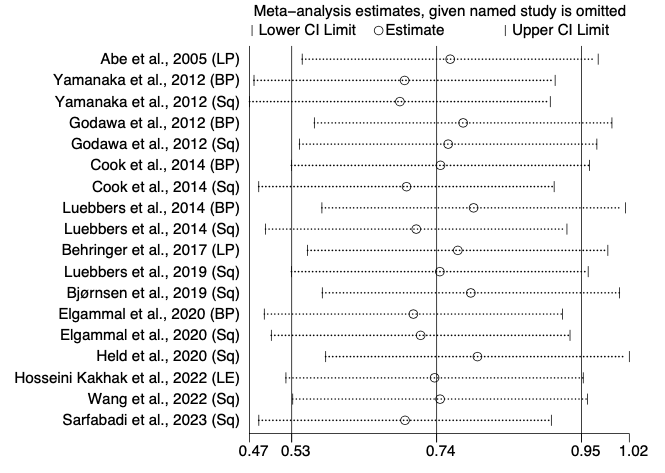

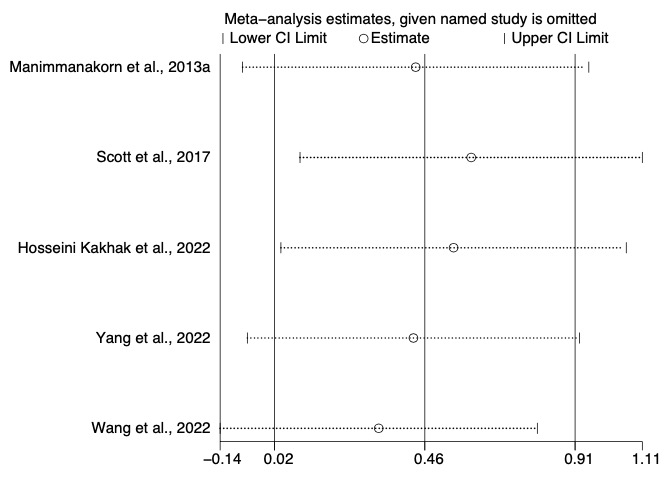


**Sprint Performance VO_2max_ Running Performance**

**_
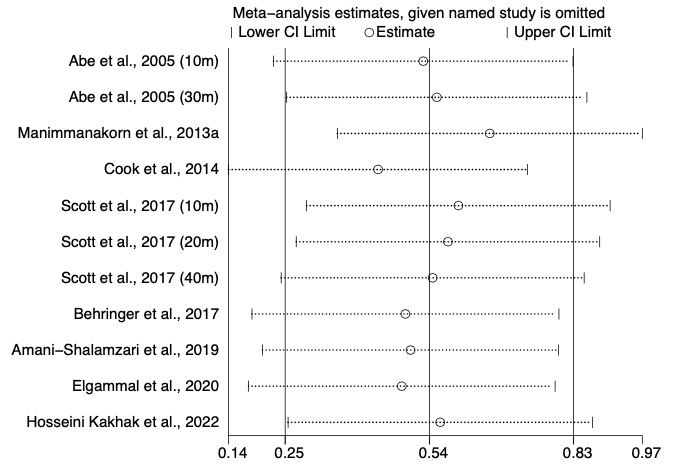

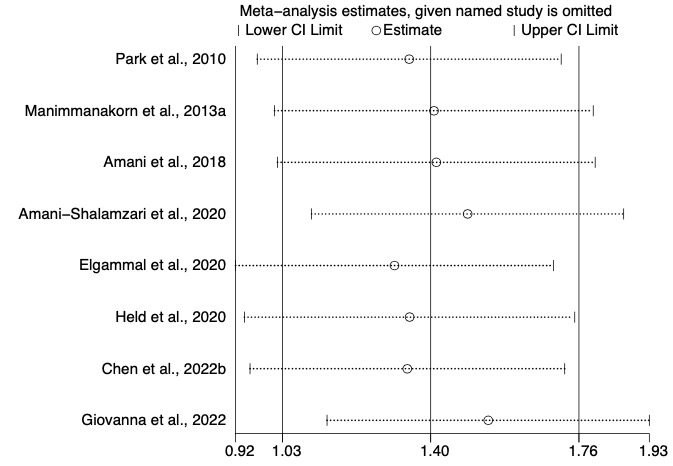

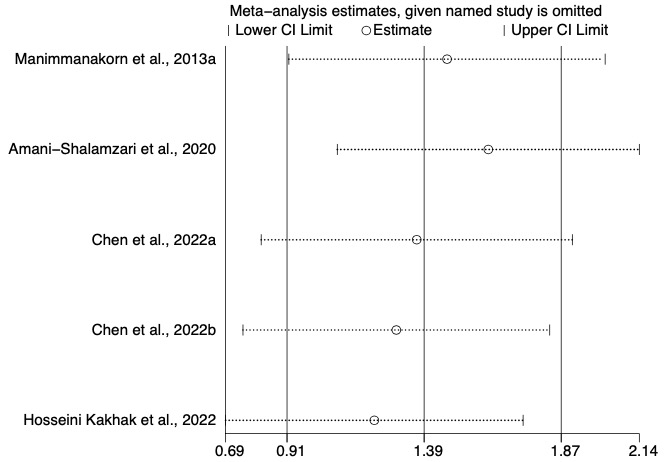
_**

**CSA Muscle Thickness Body Girths**

**
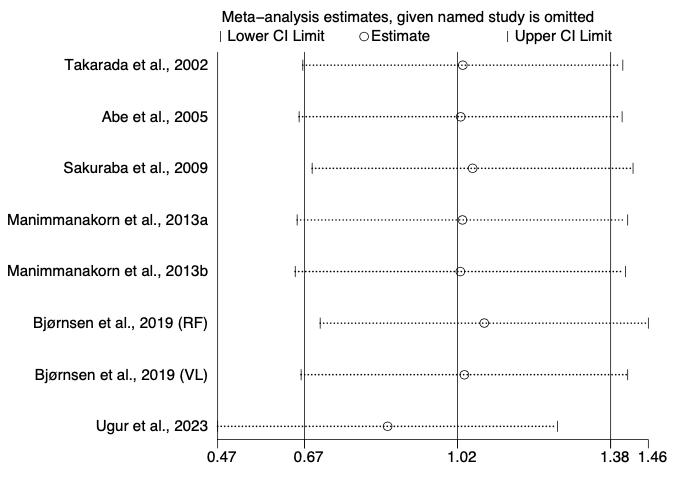

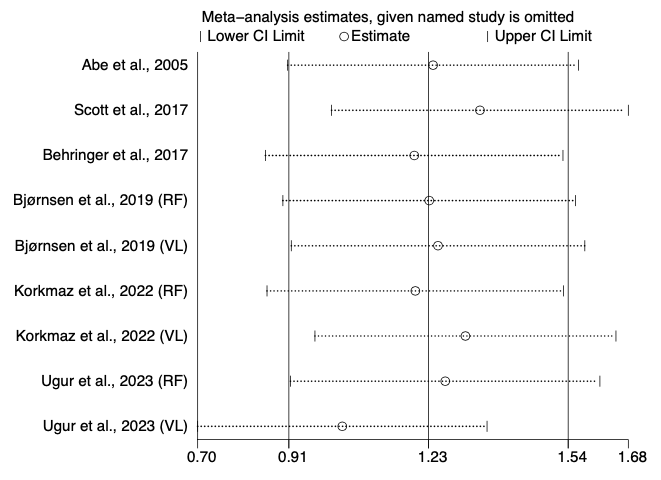
_
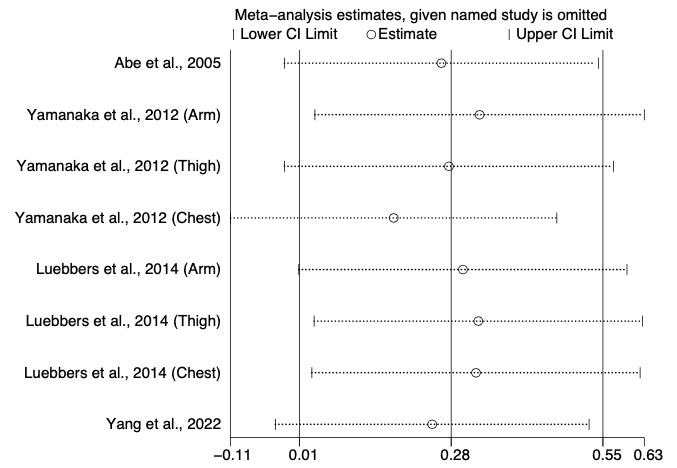
_**

**Supplementary Figure 1.** Sensitivity analysis.

**Isokinetic Strength 1RM CMJ Sprint Performance VO_2max_**


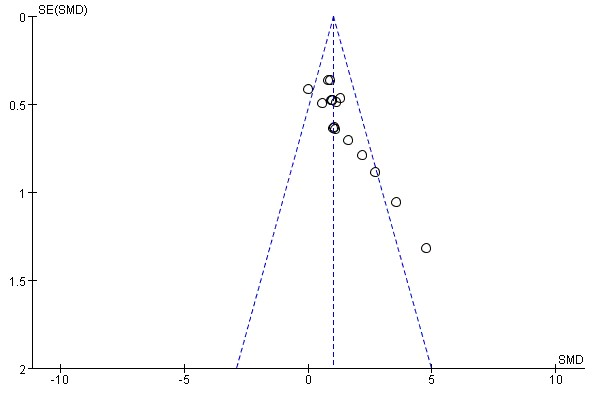

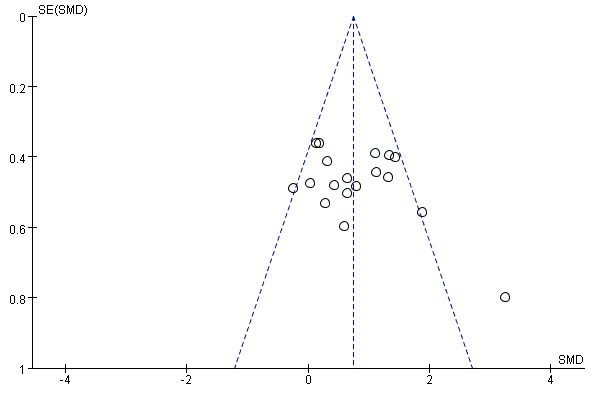

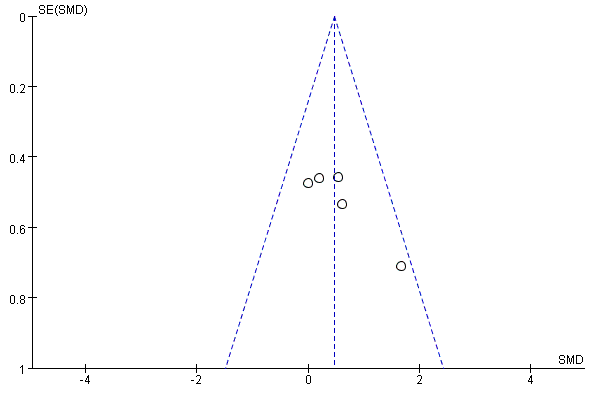

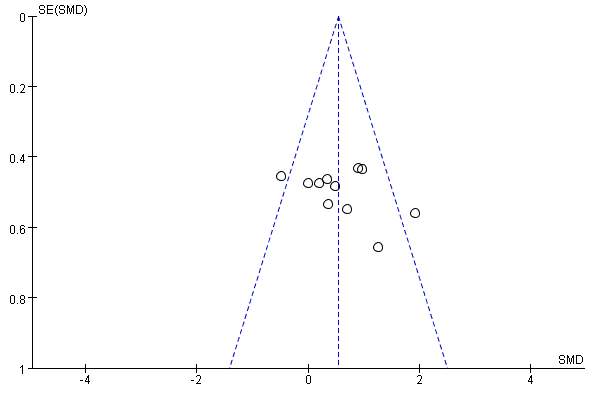

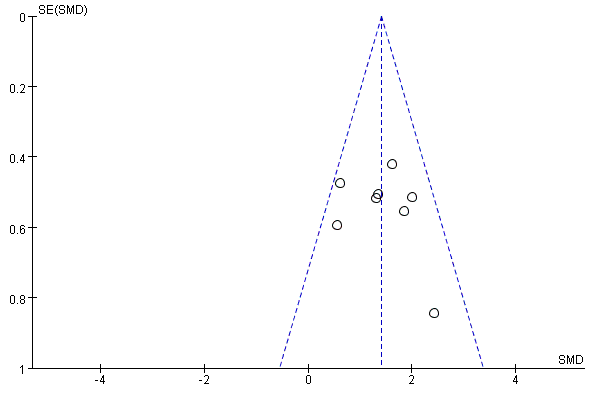


**Running Performance BM CSA Muscle Thickness Body Girths**

**
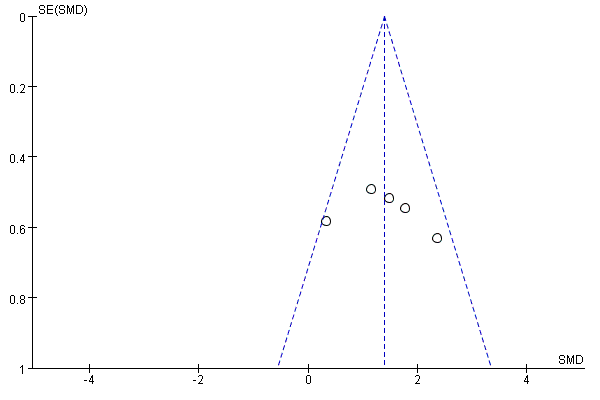

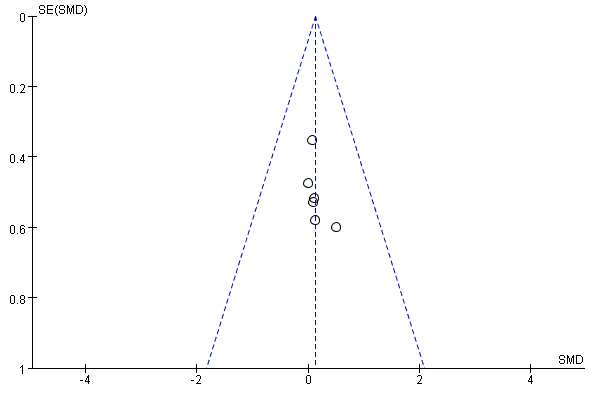
**
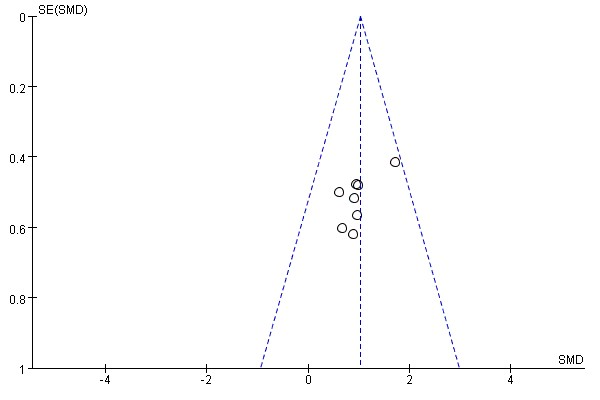

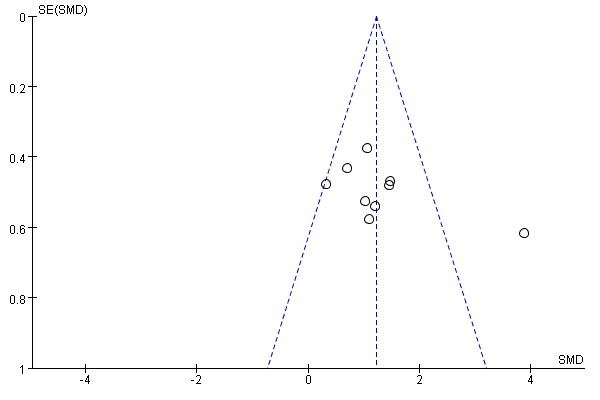
**
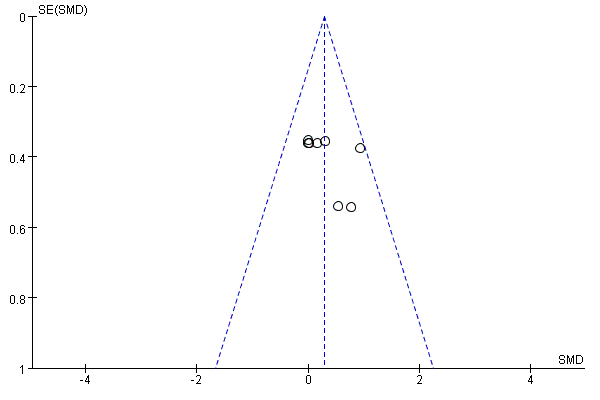
**

**Supplementary Figure 2.** Publication bias plot.
